# Synthesis of precision antibody conjugates using proximity-induced chemistry

**DOI:** 10.7150/thno.62444

**Published:** 2021-08-27

**Authors:** Yu J. Cao, Chenfei Yu, Kuan-Lin Wu, Xuechun Wang, Dong Liu, Zeru Tian, Lijun Zhao, Xuexiu Qi, Axel Loredo, Anna Chung, Han Xiao

**Affiliations:** 1 State Key Laboratory of Chemical Oncogenomics, Key Laboratory of Chemical Genomics, Peking University Shenzhen Graduate School, Shenzhen, 518055, China; 2 Department of Chemistry, Rice University, 6100 Main Street, Houston, Texas, 77005, USA; 3 Department of Biosciences, Rice University, 6100 Main Street, Houston, Texas, 77005, USA; 4 Department of Bioengineering, Rice University, 6100 Main Street, Houston, Texas, 77005, USA

**Keywords:** Antibody-drug conjugates, Bispecific antibodies, Proximity-Induced Chemistry, Site-specific conjugation, Antibody Conjugation

## Abstract

**Rationale:** Therapeutic antibody conjugates allow for the specific delivery of cytotoxic agents or immune cells to tumors, thus enhancing the antitumor activity of these agents and minimizing adverse systemic effects. Most current antibody conjugates are prepared by nonspecific modification of antibody cysteine or lysine residues, inevitably resulting in the generation of heterogeneous conjugates with limited therapeutic efficacies. Traditional strategies to prepare homogeneous antibody conjugates require antibody engineering or chemical/enzymatic treatments, processes that often affect antibody folding and stability, as well as yield and cost. Developing a simple and cost-effective way to precisely couple functional payloads to native antibodies is of great importance.

**Methods:** We describe a simple proximity-induced antibody conjugation method (pClick) that enables the synthesis of homogeneous antibody conjugates from native antibodies without requiring additional antibody engineering or post-synthesis treatments. A proximity-activated crosslinker is introduced into a chemically synthesized affinity peptide modified with a bioorthogonal handle. Upon binding to a specific antibody site, the affinity peptide covalently attaches to the antibody via spontaneous crosslinking, yielding an antibody molecule ready for bioorthogonal conjugation with payloads.

**Results:** We have prepared well-defined antibody-drug conjugates and bispecific small molecule-antibody conjugates using pClick technology. The resulting conjugates exhibit excellent *in vitro* cytotoxic activity against cancer cells and, in the case of bispecific conjugates, superb antitumor activity in mouse xenograft models.

**Conclusions:** Our pClick technology enables efficient, simple, and site-specific conjugation of various moieties to the existing native antibodies. This technology does not require antibody engineering or additional UV/chemical/enzymatic treatments, therefore providing a general, convenient strategy for developing novel antibody conjugates.

## Introduction

Antibody conjugates (e.g., antibody-drug conjugates (ADCs), bispecific small molecule-antibody (bsAb) conjugates, and others) are emerging as a new class of targeted cancer therapies that combine the targeting capability of antibodies with the exceptional cell-killing ability of toxic drugs or immune cells.^1^-^4^ By harnessing cell killing agents to the specific targeting ability of antibodies, antibody conjugates maximize the anti-tumor activity of these agents while minimizing exposure to normal tissue. Because of their exceptional potency and selectivity, eleven ADCs (Gemtuzumab ozogamicin, Brentuximab vedotin, Trastuzumab emtansine, Inotuzumab ozogamicin, Polatuzumab vedotin, Enfortumab vedotin, Trastuzumab deruxtecan, Sacituzumab govitecan, Belantamab mafodotin, Moxetumomab pasudotox, and Loncastuximab tesirine) have been approved by the US Food and Drug Administration for the treatment of various types of cancer. Production of these small molecule-antibody conjugates has relied exclusively on either the random acylation of antibody lysine residues with highly reactive esters or the random alkylation of antibody cysteine residues with maleimides.^1^,^3^,^5^ Both of these methodologies inevitably yield heterogeneous products with variable drug-to-antibody ratios (DARs) and mixed pharmacological properties, which can be challenging for the manufacturing control.^6^-^8^ Furthermore, a recent study indicates that ADC with a fixed DAR has optimal potency and safety profile.^9^ Thus, site-specific conjugation methods for producing homogeneous ADCs with constant DAR are highly desired.

Advances in antibody engineering and bioorthogonal chemistry have made it possible to prepare homogeneous site-specific antibody conjugates that exhibit enhanced stability, pharmacokinetics, and safety profiles. These next-generation antibody conjugation methods require the initial site-specific introduction of a unique reactive moiety into the antibody, followed by bioorthogonal coupling of a functional payload to the unique chemical moiety.^10^-^14^ These types of novel antibody conjugates have been prepared using several strategies. THIOMAB uses site-directed mutagenesis to introduce “hot” cysteines into the antibody as sites for conjugation.^15^ Disulfide rebridging yields antibody conjugates by reaction with cysteine thiols liberated from reduced interchain disulfides.^14^,^16^-^18^ SMARTag genetically encodes a peptide tag ready for the generation of an aldehyde-bearing formylglycine via enzymatic treatment.^19^-^21^ The site-specific labeling system introduces an unnatural sugar or noncanonical 21^st^ amino acid that enables site-specific incorporation via a distinct reactive moiety.^22^-^31^ Despite the ability to generate these homogeneous antibody conjugates, the introduction of the bioorthogonal moieties requires additional antibody engineering or chemical/enzymatic treatments that are often technically challenging and, more importantly, are likely to affect antibody folding and stability.

**Figure 1:**
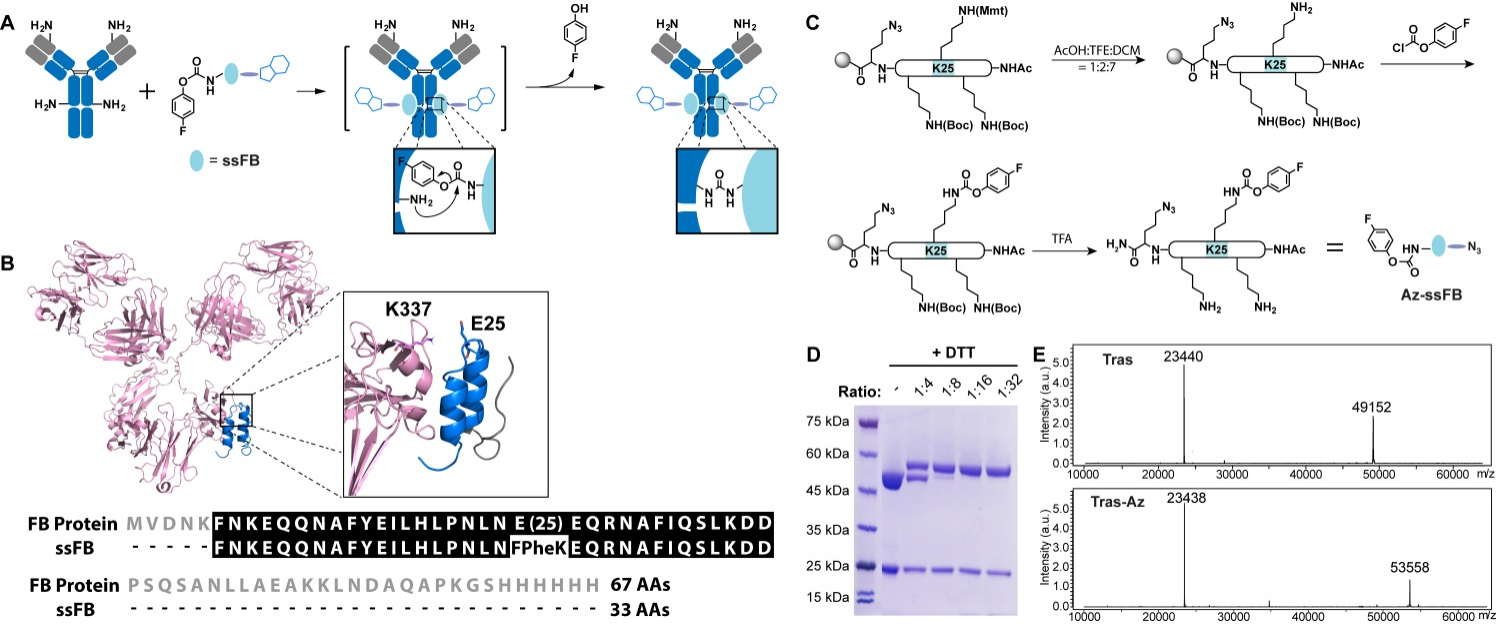
Preparation of the Tras conjugates using pClick technology. **(A)** FPheK allowing for crosslinking to a proximal lysine residue is introduced into FB peptide using solid phase synthesis. The resulting adaptive peptide (ssFB) can be used to site-specifically label native antibodies upon binding. **(B)** The structures and sequences of the B domains from *Staphylococcus* protein A (FB proteins) and ssFB used in pClick (PDB: 1FC2). **(C)** Synthesis of antibody crosslinking peptide modified with azide (Az-ssFB). **(D)** Reducing SDS-PAGE analysis of reactions of Tras with Az-ssFB at 1:4, 1:8, 1:16, and 1:32 ratios for 48 h, visualized by Coomassie staining. **(E)** Mass spectrometry analysis of Tras and Tras-Az.

Proximity-induced chemistry allows for the selective reaction between a functional group and a specific natural residue (e.g. lysine, cysteine, serine) of proteins through the complex-induced proximity effect.^32^-^39^ Coupled with mass spectrometric or other analyses, it has been widely used to capture weak and transient interactions of biomolecules.^32^-^39^ Because of its ability to modify a specific natural residue of protein, proximity-induced chemistry may afford a powerful strategy to label native antibodies without introducing unnatural moiety into the antibody. We recently developed a novel proximity-induced antibody conjugation strategy that enables the crosslinking between native antibodies and affinity proteins (**Figure 1A**).^40^-^42^ To target a specific antibody site, we previously used a 66-amino acid antibody-binding protein (FB protein) derived from *Staphylococcus aureus* protein A (**Figure 1B**).^43^ This FB protein binds to the CH2-CH3 junction of the immunoglobulin G (IgG) molecule with a dissociation constant (*K_D_*) of 10-100 µM. Furthermore, FB peptide is known to have a relatively low risk of immunogenicity.^44^ The crosslinker, 4-fluorophenyl carbamate lysine (FPheK), was genetically incorporated into the FB protein using a bioorthogonal aminoacyl tRNA synthetase (aaRS)/tRNA pair. Upon binding to the antibody, this modified FB protein formed a covalent crosslink with IgG Lys337. Despite this efficient conjugation to intact antibodies, the relatively large size and low production yield of FPheK-containing FB protein prepared by Genetic Code Expansion greatly limit the further application of this strategy.

In this study, we developed a novel proximity-induced site-specific antibody conjugation method (pClick) that is based on the proximity-induced reactivity between an affinity peptide cross-linker and a nearby antibody lysine residue (**Figure 1A**). Specifically, solid-phase peptide synthesis is used to introduce the FPheK moiety at a specific site in the affinity peptide. Upon binding to the antibody, the FPheK-containing peptide enables proximity-induced covalent attachment to a nearby antibody lysine residue. To illustrate the utility of this concept, we have prepared well-defined ADCs and bsAb conjugates. The resulting conjugates exhibit excellent cytotoxic activity against cancer cells *in vitro* and superb anti-tumor activity in mouse xenograft models.

## Results and Discussion

### Design and Optimization of Proximity-Induced Antibody Conjugation

To explore whether a shorter proximity-activated crosslinking peptide can be prepared using chemical synthesis, we examined the co-crystal structure of FB protein and the IgG Fc fragment (**Figure 1B**). The co-crystal structure suggested that the FB protein *C*-terminal loop is not involved in binding to the antibody. We, therefore, tested a 33 amino acid FB peptide with both *C*-terminal truncation and FPheK modification (Amino acid sequence of ssFB: FNKEQQNAFYEILHLPNLNXEQRNAFIQSLKDD (X=FPheK)) for its ability to bind to the antibody. To enable selective modification of this peptide with payload, an azide moiety was also introduced at the peptide *C*-terminus. As shown in **Figure 1C**, the azide-containing ssFB peptide (Az-ssFB) was synthesized using the general Fmoc-based peptide synthesis method,^45^ followed by a lysine-selective modification with 4-fluorophenyl chloroformate. To selectively introduce 4-fluorophenyl carbamate at position 25, Lys25 was first orthogonally protected with 4-methoxytrityl (MMT). After the final reaction cycle, the *N*-terminus of ssFB was deprotected and capped by acetylation. The MMT protection group could be selectively removed with 10% AcOH solution (AcOH:TFE:DCM=1:2:7), leaving the other protection groups in place.^46^ The exposed amine group of the lysine side chain was then reacted with 4-fluorophenyl chloroformate under mild basic conditions to generate the FPheK residue. The resulting peptide was cleaved, purified, refolded, and further characterized by ESI-MS analysis (**Figure S1**). To test the conjugation efficiency of Az-ssFB peptide to native antibody, we used the native trastuzumab (Tras) antibody against human epidermal growth factor receptor 2 (HER2) as a model. Different equivalents of Az-ssFB peptide were co-incubated with Tras antibody at 37 ˚C for 2 days in PBS (pH 8.5) buffer and purified on a 5K PD-10 desalting column to remove unreacted ssFB-azide peptides. The formation of conjugate (Tras-Az) was confirmed by SDS-PAGE analysis (**Figure 1D**). To our delight, the conjugation reaction with 16 equivalents of Az-ssFB peptide produced the desired conjugate with a yield greater than 95%. ESI-MS analysis of the final conjugate product (Tras-Az) indicated no mass shifts for the light chain of Tras, but a mass difference of 4406 Da, in agreement with the calculated addition of Az-ssFB peptide (**Figure 1E**).

### Synthesis of Site-Specific ADC using pClick

**Figure 2:**
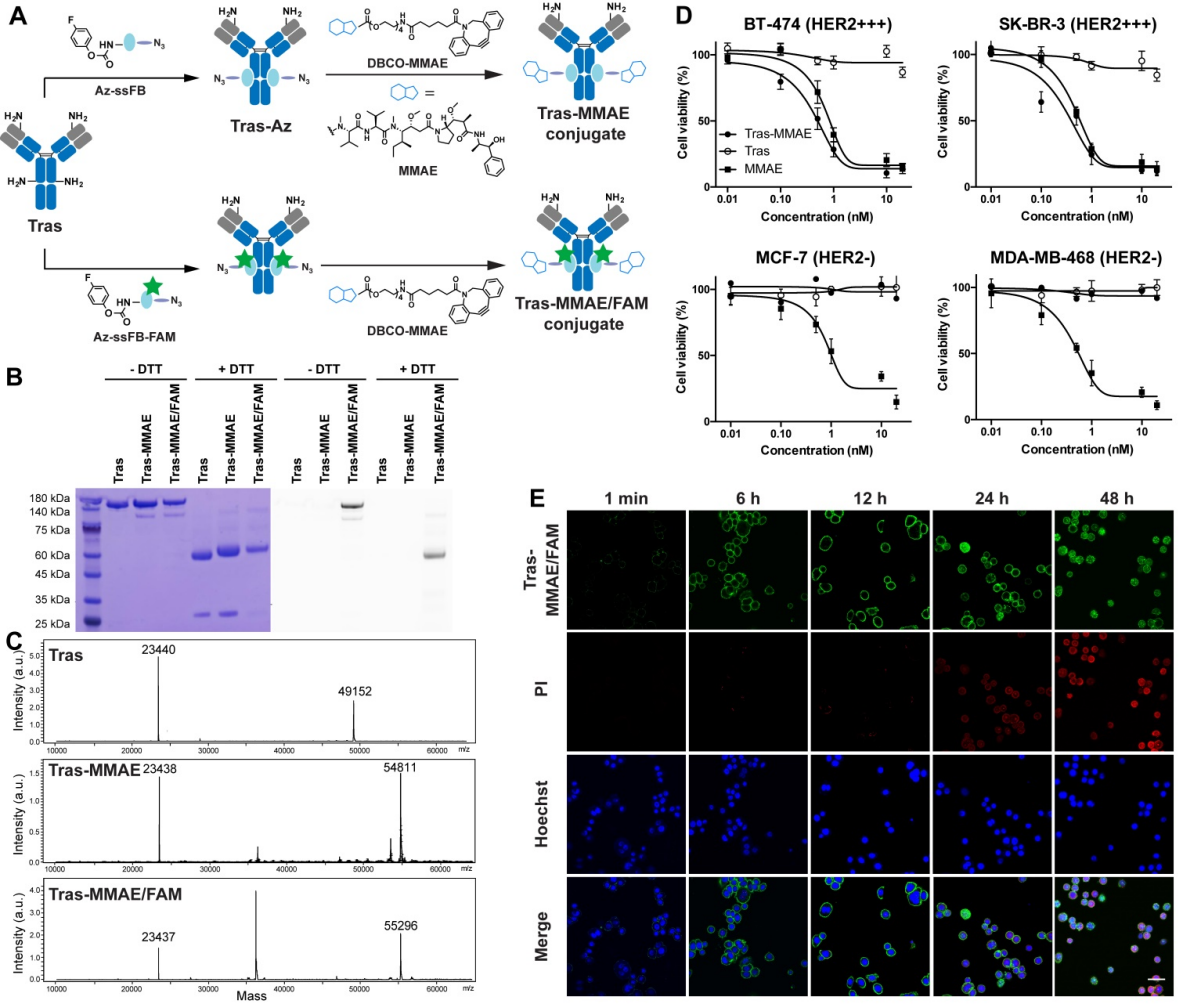
** (A)** Preparation of homogeneous ADCs using pClick technology. Tras antibody was first modified with Az-ssFB or Az-ssFB-FAM using pClick technology, and then labeled with DBCO-MMAE using copper-free click chemistry to yield Tras-MMAE and Tras-MMAE/FAM conjugates, respectively. **(B)** SDS-PAGE analysis of Tras, Tras-MMAE, and Tras-MMAE/FAM under reducing and non-reducing conditions, visualized by Coomassie staining (left) and UV transillumination (right). **(C)** ESI-MS analysis of Tras, Tras-MMAE, and Tras-MMAE/FAM. The peak of 36 kDa corresponds to the PNGase F enzyme used in the deglycosylation reaction. **(D)** *In vitro* cytotoxicity of MMAE, Tras, and Tras-MMAE, against HER2-positive and -negative cancer cells. **(E)** The internalization of Tras-MMAE/FAM was monitored using fluorescent imaging. SK-BR-3 cells were incubated with 10 nM Tras-MMAE/FAM for 1 min, 6, 12, 24, 48 h and stained with PI (red) and Hoechst stain (Blue). Scale bar = 50 µm.

After establishing the efficiency of the conjugation reaction between Az-ssFB peptide and antibodies, we synthesized an ADC with a non-cleavable linker for treating breast cancer by site-specifically conjugating the Tras antibody to monomethyl auristatin E (MMAE), a potent drug that interferes with cell division by blocking tubulin polymerization (**Figure 2A**).^47^ Using dibenzylcyclooctyne (DBCO)-linked PEG, we synthesized DBCO-MMAE with a DBCO group added at the *N*-terminus of MMAE. The azide-containing Tras antibody (Tras-Az) was coupled to 20 equivalents of DBCO-MMAE overnight in PBS buffer using copper-free click chemistry reaction, followed by purification on a PD-10 desalting column to remove unreacted small molecules. SDS-PAGE and ESI-MS spectrometry analysis revealed a 5659 Da peak shift between unconjugated Tras antibody heavy chain (49152 Da) and Tras-MMAE conjugate heavy chain (54811 Da), indicating an overall yield of more than 90% for conjugation of two MMAE molecules per antibody (**Figure 2B**-**C**). These results confirm that a homogeneous antibody-drug conjugate containing two toxins at a defined position (one per heavy chain) can be produced by pClick technology. To ensure that the introduction of MMAE has minimum perturbance of the binding of the antibody, we carried out a flow cytometry study. To our delight, modification of antibody using pClick doesn't exhibit altered antigen binding (**Figure S2**). *In vitro* cytotoxicity of the Tras-MMAE conjugate prepared using pClick was then evaluated using HER2-expressing breast cancer cells (SK-BR-3 and BT-474) and HER2-negative cells (MCF-7 and MDA-MB-468). Consistent with the specificity of the Tras antibody, the Tras-MMAE conjugate efficiently kills SK-BR-3 and BT-474 cells with EC50 values of 0.11 ± 0.11 nM and 0.75 ± 0.54 nM, respectively, but has little effect on MCF-7 and MDA-MB-468 cells (EC_50_ > 500 nM) (**Figure 2D**). In contrast, unconjugated auristatin exhibits similar cytotoxic activities against both HER2-positive and HER2-negative cells (**Figure 2D**). Compared to previously reported trastuzumab-MMAE conjugates with a similar DAR, Tras-MMAE prepared by pClick exhibits similar cytotoxicity activities against HER2-positive/negative cell lines.^48^,^49^

### Preparation and Characterization of Multifunctional ADC using pClick

Having established a robust conjugation strategy for ADC preparation, we next explored the utility of the pClick technology for preparing multifunctional ADCs with capabilities for both imaging and drug delivery. To develop this methodology, we first used solid-phase peptide synthesis to couple 5/6-carboxyfluorescein (FAM)-Lysine to the *N*-terminus of the Az-ssFB peptide. Following on-resin generation of FPheK, the resulting Az-ssFB-FAM peptide was cleaved from the resin and purified by HPLC (**Figure S1**). The resulting peptide was refolded and added to the native Tras antibody and incubated for 48 h in pH 8.5 PBS buffer. Upon completion of the reaction, the resulting conjugate was purified on a PD-10 desalting column, followed by the reaction with DBCO-MMAE using a copper-free click reaction. This two-step conjugation procedure generated Tras-MMAE/FAM with a yield greater than 98%, as determined by SDS-PAGE and ESI-MS analysis (**Figure 2B-C**). The Tras-MMAE/FAM band was only observed under UV transillumination, indicating successful incorporation of the fluorophore into the heavy chain of Tras (**Figure 2B**).

With the fluorophore-labeled ADC in hand, we evaluated its uptake and ability to induce apoptosis in HER2-positive SK-BR-3 cells (**Figure 2E**). Cells were incubated with 10 nM Tras-MMAE/FAM for 1 min, 6 h, 12 h, 24 h, 48 h. Cell surface-associated green fluorescence can be detected immediately after the addition of Tras-MMAE/FAM conjugate. After 6 h incubation, intracellular fluorescence can also be detected, indicative of significant antibody internalization. To correlate Tras-MMAE/FAM internalization with drug-induced apoptosis, cell viability was monitored using propidium iodide (PI) red fluorescence. Within 12 h, only a negligible PI signal could be observed, while the apoptosis-specific signal became prominent after 24 h. Furthermore, we have compared the internalization rates of site-specific and non-site-specific antibody conjugates. Antibody conjugates prepared by pClick didn't exhibit a significantly different internalization rate compared with an antibody labeled by *N*-hydroxysuccinimidyl esters (**Figure S3**). Thus, the precise dual-labeling of antibodies with a drug and a fluorescent probe (or alternatively, a chelator for radiolabeling) affords an efficient platform for evaluating the therapeutic and diagnostic properties of ACDs in pre-clinical model systems.

### Synthesis and Efficacy of Bispecific Antibody-Small Molecule Conjugates

**Figure 3:**
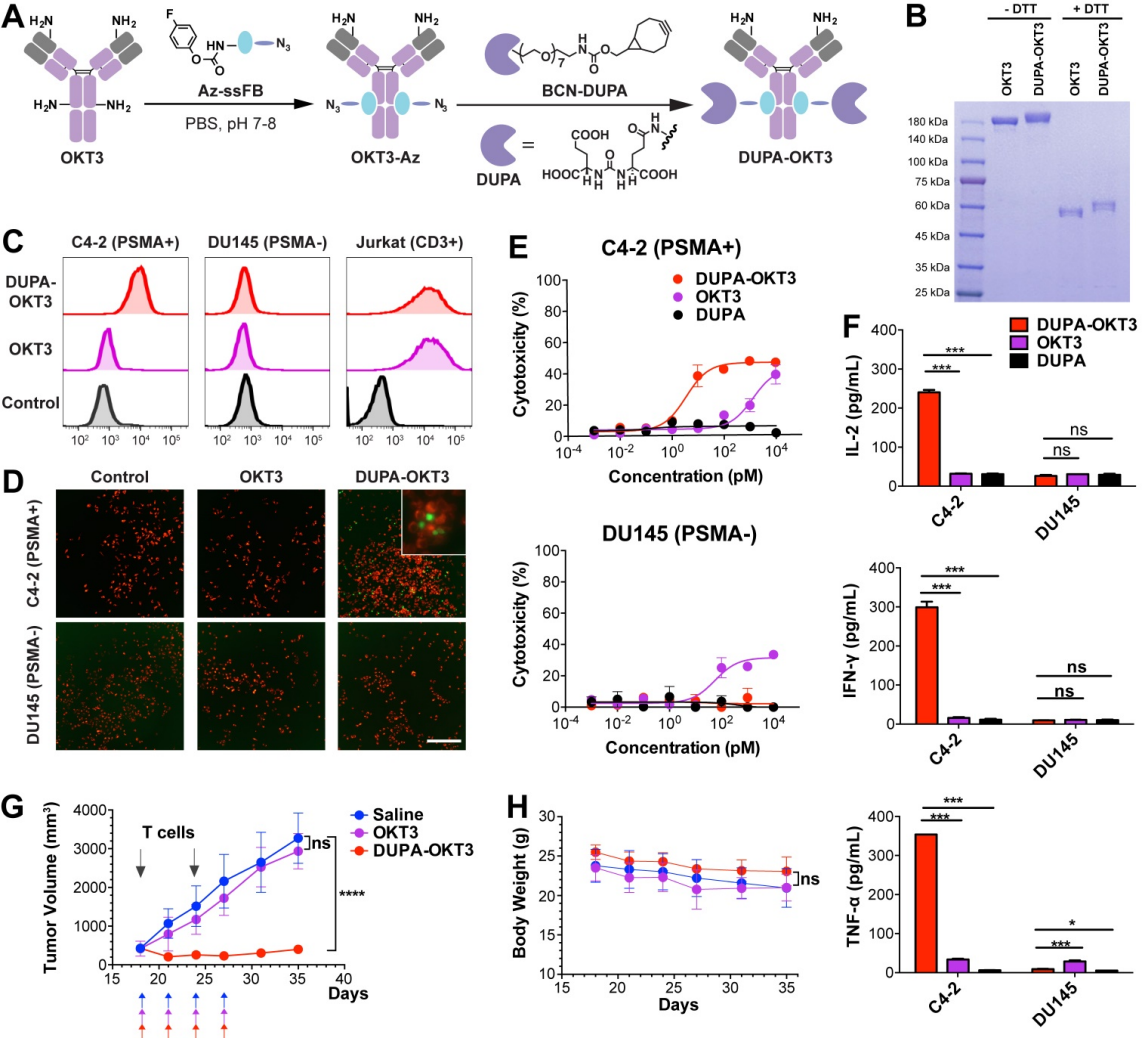
** (A)** Preparation of bispecific small molecule-antibody conjugates. OKT3 antibody was first modified with Az-ssFB using pClick technology, and then labeled with BCN-DUPA using copper-free click chemistry. **(B)** SDS-PAGE analysis of OKT3 and DUPA-OKT3 under reducing and non-reducing conditions. **(C)** Flow cytometry based binding assay. Primary antibody (100nM) was incubated C4-2 (PSMA+), DU145 (PSMA-) and Jurkat (CD3+) cell line and then detected by a secondary antibody (anti-mouse IgG-APC). **(D)** Fluorescence microscopy images of the interaction between C4-2 (red) cells and Jurkat cells (green) in the presence of the DUPA, OKT3 and the DUPA-OKT3 conjugate. **(E)** cytotoxic activity of PBMCs against PSMA-positive C4-2 cells and PSMA-negative DU145 cells in the presence of indicated concentrations of different drugs. Cytolytic activity was determined by measuring of the amount of lactate dehydrogenase (LDH) released into cultured medium. **(F)** Cytokine levels in the cultures consisting of different cancer cells, purified T cells, and 10nM of different drugs (DUPA, OKT3, and DUPA-OKT3).** (G)** *In vivo* efficacy comparison of DUPA-OKT3 and OKT3 in human prostate cancer xenograft models. Eighteen days after subcutaneous implantation of 2×10^6^ C4-2 prostate cancer cells in 50% Matrigel, male NCG mice were injected twice with 25×10^6^ activated T cells six days via I.P. On the same day of initial T-cell infusion, mice were treated intravenously with four doses of DUPA-OKT3, OKT3 or saline every 2 days. Black arrows indicate the time of activated T cells injections of treatment with specific therapeutics. **(H)** Body weight change of tumor-bearing mice over time. Error bars represent SD. ****= p<0.0001, and ns=p>0.05 (not significant) were calculated using the Student's t-test.

As a model for demonstrating the utility of pClick in preparing bispecific antibodies, we used a urea-based inhibitor (DUPA) of glutamate carboxypeptidase II (PSMA)^50^ and the FDA-approved anti-CD3 antibody (muromonab, OKT3). The membrane protein PSMA is over-expressed in all stages of prostate cancer and has been recently identified as a medically relevant target for the diagnosis and therapy of this disease. Among all small molecule inhibitors of PSMA, DUPA exhibits an especially high degree of affinity (*Ki* = 8 µM) and selectivity.^51^-^53^ Thus, crosslinking PSMA-expressing prostate cancer cells and CD3-positive T cells using bispecific DUPA-muromonab antibody (DUPA-OKT3) is likely to induce potent T cell activation in a manner that is dependent on their binding to antigen-positive tumor cells. To prepare the DUPA-OKT3 conjugate, the OKT3 antibody was first site-specifically modified with Az-ssFB peptide using pClick technology, followed by the copper-free click reaction with bicyclo[6.1.0]nonyne (BCN)-DUPA (**Figure 3A**). Successful preparation of the DUPA-OKT3 conjugate was verified by SDS-PAGE (**Figure 3B**) and ESI-MS (**Figure S4**).

We next used flow cytometry to assess the binding of OKT3 and DUPA-OKT3 to the PSMA-expressing prostate cancer cell line C4-2, to the PSMA-negative prostate cancer cell line DU145, and to the CD3-positive Jurkat T cell line (**Figure 3C** and **S5**). The specificity of DUPA-OKT3 binding to PSMA-positive C4-2 cells relative to PSMA-negative DU145 cells is reflected by the C4-2 binding index of 125. DUPA-OKT3 also exhibited excellent binding to CD3-positive Jurkat cells (relative binding index of 255 to Jurkat), comparable to that seen with parental OKT3 antibody (relative binding index of 251). The ability of DUPA-OKT3 to crosslink PSMA-expressing prostate cancer cells and CD3-positive T cells was further evaluated using fluorescence imaging (**Figure 3D**). Specifically, C4-2 or DU145 cells were stained with Mito Tracker Red (Invitrogen, red fluorescence), and Jurkat cells were stained with carboxyfluorescein succinimidyl ester (CFSE, Biolegend, green fluorescence). The labeled Jurkat cells were incubated with 100 nM DUPA, OKT3, or DUPA-OKT3 in RPMI media at 4 °C for 30 min, and excess drugs were removed by washing prior to mixing with labeled C4-2 or DU145 cells. In the DUPA-OKT3 incubated samples, significantly more Jurkat cells were bound to the C4-2 cells compared to cells incubated with either DUPA or OKT3, confirming the ability of the DUPA-OKT3 conjugate to mediate cell-cell crosslinking (**Figure 3D**).

To assess the ability of DUPA-OKT3 to selectively direct T cells to PSMA-expressing cancer cells in a functionally significant manner, we performed a cytotoxicity assay using C4-2 and DU145 cancer cells in the presence of human peripheral blood mononuclear cells (PBMCs). As shown in Figure 3E, DUPA-OKT3 exhibited excellent T cell-dependent cytotoxic activity against PSMA-expressing C4-2 cancer cells (EC_50_ value of 3.54 ± 0.11 pM) compared to DU145 cells. In contrast, OKT3 itself induced antigen-independent cytotoxic activity after 24 h against both C4-2 and DU145 cells in the presence of PBMCs. Importantly, the site-specific conjugation of OKT3 with DUPA enhances the specific T cell interaction with target cells, avoiding possible off-target effects of using unmodified OKT3. Furthermore, treatment of mixed cultures of C4-2 cells and T cells with 100 or 10 nM DUPA-OKT3 resulted in a significant upregulation of both T-cell activation markers (CD25 and CD69, **Figure S6**) and cytokine production (IL-2, IFN-γ, TNF-α, **Figure 3F** and** S7**), compared with levels of these entities seen with DU145 cells and T cells treated separately. Taken together, these data confirm that DUPA-OKT3 prepared by pClick strategy induces T cell activation in an antigen-dependent manner, resulting in potent and selective *in vitro* activity against PSMA-expressing cancer cells.

To further evaluate the *in vivo* efficacy of DUPA-OKT3, xenograft tumors were established by subcutaneous implantation of PSMA-expressing C4-2 cells in male NCG mice. When tumor volumes reached 400 mm^3^, mice were infused intraperitoneally with human T cells, followed by intravenous administration of DUPA-OKT3, OKT3 (both at 1 mg/kg), or saline every other day. Shortly after treatment was initiated, significant tumor shrinkage was observed only in the DUPA-OKT3 group, whereas the OKT3 and saline groups both exhibited rapid tumor growth. After 10 days of treatment, tumor growth was monitored for an additional 10 days in the absence of additional treatment. During this extended time period, significant tumor regrowth was not observed in DUPA-OKT3 treatment group compared to the control groups treated with saline or OKT3 (**Figure 3G**). These results demonstrate that DUPA-OKT3 produced via the pClick methodology is efficacious against established tumors in mice. Significantly, no drug-induced toxicity was observed in the mice treated with DUPA-OKT3, as evidenced by the absence of body weight loss (**Figure 3H**). Cytokine production *in vivo*, especially that of IFN-γ, was generally correlated with the antitumor efficacy of redirected T cells. As shown in **Figure S8**, mice dosed with 1 mg/kg of DUPA-OKT3 exhibited a significant increase in serum IFN-γ compared to the saline-treated group. Our results suggest that the pClick strategy will facilitate the application of bsAb therapy to solid tumors. Current methods for producing these reagents are very labor-intensive and time-consuming.

### Summary

In summary, we have developed a new proximity-induced antibody conjugation method (pClick) for the construction of homogeneous antibody conjugates. We have demonstrated the utility of this platform by preparing well-defined antibody-drug and bispecific antibody conjugates. Native anti-HER2 antibody was site-specifically coupled to either the microtubule toxin MMAE or both MMAE and the imaging probe without the use of antibody engineering or additional post-treatments. The resulting ADC conjugates exhibit excellent cytotoxicity and selectivity against HER2-expressing cancer cells. The well-defined multifunctional ADC provides a direct and general strategy for probing the distribution and internalization of a variety of ADCs. Furthermore, using pClick technology, we synthesized a bispecific antibody that can efficiently redirect T cells to target PSMA-expressing prostate cancers, inducing cancer cell cytotoxicity at picomolar concentrations *in vitro*. In mouse xenograft models, this bispecific antibody produces significant inhibition of prostate tumor growth in the absence of off-target toxicity, demonstrating potency comparable to the best current examples in the field.

The ability to site-specifically label antibodies with payloads possessing different chemical, physical, and biological properties provides the means for preparing antibody conjugates with a variety of enhanced capabilities. Existing methods for labeling antibodies require extensive antibody engineering and additional chemical or enzymatic treatments that result in low yields and reduced antibody stability. In contrast, the pClick technology developed in our study enables efficient site-specific covalent bond formation between payloads and native antibodies without the need for antibody engineering or additional steps. To achieve the site-specific conjugation on other sites of antibodies, more antibody-binding peptides are currently explored for use in the pClick conjugation technology. pClick provides a simple and general way of precisely coupling functional payloads to antibodies, providing new paths toward cancer diagnosis and therapy.

## Experimental Section

### Materials

Unless otherwise noted, the chemicals and solvents used were of analytical grade and were used as received from commercial sources. Fmoc-Ala-OH, Fmoc-Asp(OtBu)-OH, Fmoc-Glu(OtBu)-OH, Fmoc-Phe-OH, Fmoc-His(Trt)-OH, Fmoc-Ile-OH, Fmoc-Lys(Boc)-OH, Fmoc-Lys(MMT)-OH, Fmoc-Leu-OH, Fmoc-Asn(Trt)-OH, Fmoc-Pro-OH, Fmoc-Gln(Trt)-OH, Fmoc-Arg(Pbf)-OH, Fmoc-Ser(tBu)-OH, Fmoc-Tyr(tBu)-OH and Fmoc-Lys(N_3_)-OH were purchased from Chemimpex. Fmoc-Lys(5/6-FAM)-OH was purchased from Anaspec. Trifluoroacetic acid (TFA), 1-[Bis(dimethylamino)methylene]-1H-1,2,3-triazolo[4,5-b]pyridinium 3-oxide hexafluorophosphate (HATU) and *N.N*-Diisopropylethylamine (DiEA) were purchased from Oakwood Chemical. Rink Amide MBHA LL resin was purchased from EMD Millipore. Anisole and TIPS (triisopropylsilane) were purchased from Chemimpex. *N.N*-dimethylformamide (DMF), dichloromethane (DCM), HPLC-grade acetonitrile, diethyl ether, and HPLC-grade acetonitrile were obtained from Fisher Chemicals. Human, IgG1 trastuzumab (Herceptin) is a gift from Genentech, Inc. PNGase F was purchased from New England Biolabs to remove the glycans before ESI-MS analysis. Before conjugation, the antibody affinity peptides were dialyzed by Spectra/Por® G, MWCO: 500-1000 Da, Volume: 1 mL. NuPAGE 4-12 % Bis-Tris Gel was purchased from Invitrogen. SDS-PAGE Sample Loading Buffer [6x] was purchased from G-BIOSCIENCES. PM2500 ExcelBand 3-color Regular Range Protein Marker was purchased from SMOBIO.

### HPLC Purification of peptide crudes

Each peptide sample (20 mg/mL in water) was analyzed using Agilent HPLC systems with 1260 infinity II Quaternary Pump (Agilent: G7111B). (Column: Agilent 5 Prep-C18 100 × 21.2 mm) The elution conditions for peptides were as follows: mobile phase A= 0.1% formic acid water; mobile phase B= 0.1% formic acid in acetonitrile; gradient 0-2.1 min, 5-20% B; 2.1-32 min, 20-60% B; 32-32.1 min, 60-100% B; flow rate= 5 mL/min.

### ESI-MS analysis

The peptides and antibody conjugates were analyzed using a single quadrupole mass spectrometer (Agilent: G7129A) coupled with 1260 infinity II Quaternary Pump (Agilent: G7111B). (Column: Agilent ZORBAX 300SB-C8 5 μm 4.6×150 mm; Pursuit 5 Diphenyl 150×2.0 mm) The elution conditions for peptides were as follows: mobile phase A= 0.1% formic acid water; mobile phase B= 0.1% formic acid in acetonitrile; gradient 0-0.1 min, 10-15% B; 0.1-8 min, 15-65% B; 8-8.5 min, 65-10% B; flow rate= 1 mL/min. The elution conditions for antibody conjugates were as follows: mobile phase A= 0.1% formic acid water; mobile phase B= 0.1% formic acid in acetonitrile; gradient 0-0.1 min, 10-15% B; 0.1-8 min, 15-50% B; 8-8.1 min, 50-10% B; flow rate= 0.5 mL/min. The absorbance was measured at 280 nm. Automatic data processing was performed with MassHunter BioConfirm software (Agilent) to analyze the intact and reduced MS spectra.

### SDS-PAGE analysis

The intact and reduced antibody samples were analyzed using Invitrogen NuPAGE 4-12% Bis-Tris gel. 20 μL 0.2 mg/mL antibody samples were mixed with 4 μL 6× sample loading dye before loading into the gel. The gel was running under 160 V in MES buffer for 35 min and stained using Coomassie blue buffer. The gel images were taken by Amersham Imager 600 and analyzed by ImageQuanTL software.

### General method of peptide synthesis

Solid-phase synthesis of Az-ssFB-FAM and Az-ssFB peptide was performed by following the protocol of Fmoc-based peptide synthesis. Amino acid sequence: X'FNKEQQNAFYEILHLPNLNXEQRNAFIQSLKDDPS(AzK) (X=MMT-Lys, AzK=N_3_-Lys, X'=(5/6-FAM)-Lys). Rink Amide MBHA LL resin was used as the solid support. Fmoc protection group was removed after each coupling by 25% piperidine in DMF and washed with DMF. For the coupling, Fmoc-protected amino acid was mixed with HATU (4 equivalents), DiEA (6 equivalents), and the resin in DMF. The N-terminus of the last amino acid was capped by acetic anhydride in DCM.

To incorporate FPheK into the peptide, the MMT protection group was selectively removed by 10 % acetic acid (AcOH:TFE:DCM=1:2:7) and washed with DCM. 2 equivalents of 4-fluorophenyl chloroformate and 4 equivalents of DiEA were mixed with the resin in DCM for FPheK formation and washed with DCM.

After the reaction was completed, the resin was treated with 95% TFA/scavengers (water, anisole, triisopropyl silane) for 3 h to cleave the peptide from the resin, remove and quench all protection groups. The peptide was then precipitated by ice-cold ether, purified by HPLC and lyophilized.

### Construction of Tras-MMAE

20 equivalents of Az-ssFB peptide (0.4 mM) were mixed with Tras (0.5 mg/mL in PBS) at 37 ˚C for two days. The Tras-Az conjugate was purified via a PD-10 desalting column. 40 equivalents of DBCO-MMAE were added at RT overnight. Finally, Tras-MMAE was purified via a PD-10 desalting column and characterized by ESI-MS.

### Construction of Tras-MMAE/FAM

20 equivalents of Az-ssFB-FAM peptide (0.4 mM) were mixed with Tras (0.5 mg/mL in PBS) at 37 ˚C for two days. The resulting conjugate was purified via a PD-10 desalting column. 40 equivalents of DBCO-(PEG)_4_-MMAE was added at RT overnight for strain-promoted alkyne-azide cycloaddition (SPAAC) reaction. Finally, Tras-MMAE/FAM was purified via a PD-10 desalting column and characterized by ESI-MS.

### Construction of DUPA-OKT3 bsAb

16 equivalents of Az-ssFB peptide (2.4 mg, 2 mg/mL in PBS 8.5) were added to OKT3 (5 mg, 1 mg/mL in PBS pH 8.5) and the reaction was incubated at 37 ˚C for 48 h. The resulting OKT3 conjugate was purified by Amicon® Ultra-15 Centrifugal Filter (30 kDa) and buffer-exchanged to PBS pH 7.4. BCN-DUPA (10 mM, 30 equivalents) was added to the purified conjugate and the reaction was incubated at 37 ˚C for 24 h. Finally, the DUPA-OKT3 bsAb conjugate was purified via a PD-10 desalting column and characterized by ESI-MS.

### Binding activity by flow cytometry

The binding activity of ADC was investigated by flow cytometry. Cancer cells (1 × 10^5^) were re-suspended and stained with 30 μg/mL Tras conjugates for 30 min at 4 ˚C. After staining, cells were washed twice with PBS and fluorescence intensity was determined using a BD FACSVerse (BD Biosciences).

The binding activity of DUPA-OKT3 and parental antibody were determined by flow cytometry using human prostate cancer cells (C4-2 and DU145) and human T cell line (Jurkat). Cells were incubated with primary antibodies (100 nM) for 1 h at 4 °C. After several washes, cells were incubated with Alexa Fluor 647-conjugated anti-mouse IgG antibody (Thermo Fisher Scientific) prior to data acquisition on an Attune NxT Flow Cytometer (Thermo Fisher Scientific). All flow cytometry data were analyzed with FlowJo 10.0.6 (Treestar).

### Analysis of internalization and apoptosis

The cancer cell lines were grown to about 80% confluency in 8-well confocal imaging chamber plates. The cells were incubated with 10 nM antibodies at different time points. The cells were washed twice with PBS and incubated with propidium iodide at room temperature in the dark for 5 min. Then the cells were stained with Hoechst 33342 (Cat No: H1399, Life TechnologiesTM.) for 5 min. Cells were then washed three times with PBS (pH 7.4) and used for confocal imaging. Confocal fluorescence images of cells were obtained using a Nikon A1R-si Laser Scanning Confocal Microscope (Japan), equipped with lasers of 405/488/561/638 nm.

### Conjugation formation assay

To demonstrate the simultaneous binding of DUPA-OKT3 to CD3 and PSMA, we visualized the frequency of cancer cell-T cell interaction in the presence of bsAbs. Briefly, cancer cells and T cells were stained with MitoTracker Red (Invitrogen) and carboxyfluorescein succinimidyl ester (CFSE, Biolegend), respectively. Labeled cells were mixed at a 1: 1 ratio with 100 nM bsAbs and incubated at 37 °C for 4 h. Non-conjugated cells were removed before imaging on a Cytation 5 Cell Imaging Multi-Mode Reader (BioTek). To demonstrate the simultaneous binding of DUPA-OKT3 to CD3 and PSMA, we visualized the frequency of cancer cell-T cell interaction in the presence of bsAbs. Briefly, cancer cells and T cells were stained with MitoTracker Red (Invitrogen) and carboxyfluorescein succinimidyl ester (CFSE, Biolegend), respectively. Labeled cells were mixed at a 1: 1 ratio with 100 nM bsAbs and incubated at 37 ˚C for 4 h. Non-conjugated cells were removed before imaging on a Cytation 5 Cell Imaging Multi-Mode Reader (BioTek). The conjugation formation efficiency was calculated by: % efficiency = (number of CFSE cells conjugated with MitoTracker Red cells) / (Total MitoTracker Red cells) × 100.

### *In vitro* cytotoxicity assay

To detect the cytotoxicity of molecules, SK-BR-3, BT-474, MCF-7 and MDA-MB-468 were plated in 96-well plates at ∼5,000 cells in 100 μL per well in respective culture media and incubated overnight. The culture medium was then removed, replaced by different concentrations of MMAE, Tras and Tras-MMAE dissolved in the culture medium, and then incubated for 3 days. 20 μL of MTT solution (5 mg/mL) was then added to each well and incubated for another 4 h. Medium was aspirated and 150 μL DMSO was added to each well. The absorbance at 570 nm was measured by the microplate reader (Infinite M Plex by Tecan) to quantify living cells.

Cytotoxicity assays of bsAb were performed using human peripheral blood mononuclear cells (PBMCs) as effector cells, and prostate cancer cells as target cells. PBMCs were isolated from healthy donors by Ficoll density gradient centrifugation using standard procedures, and incubated in tissue culture flasks for > 3 h to remove adherent cells. Target cells (1 × 10^4^ cells) were mixed with PBMCs (1 × 10^5^ cells) and incubated with different concentrations of drugs for 24 h at 37 ˚C and 5% CO_2_. Cytotoxic activity was determined by measuring lactate dehydrogenase (LDH) levels in the cultured supernatant using the Cytotox-96 nonradioactive cytotoxicity assay kit (Promega). Percent cytotoxicity was calculated by: % cytotoxicity = [(absorbance experimental - absorbance spontaneous average)/(absorbance maximum killing average - absorbance spontaneous average)] × 100.

### T cell activation analysis

To selectively monitor the activation of T cells, the purified T cells were labeled with carboxyfluorescein succinimidyl ester (CFSE, Biolegend) as per manufacturer's protocol, and further incubated with target cells in the presence of bsAbs or parental antibodies. After 24 h, cells were labeled with pERcp/Cy5.5-conjugated anti-human CD25 and APC-conjugated anti-human CD69 (Biolegend), and analyzed by flow cytometry. The release of IL-2, IFN-γ, and TNF-α in the cultured supernatant was measured by enzyme-linked immunosorbent assay (ELISA) kit (Thermo Fisher Scientific). Results are shown as mean of duplicated samples ± SD.

### *In vivo* efficacy study

All efficacy studies were conducted with 6- to 8-week-old male NOD-Prkdcscid Il2rg null (NCG) mice (Nanjing Biomedical Research Institute of Nanjing University, Nanjing, China). Human prostate cancer cells C4-2 were used to evaluate the *in vivo* efficacy of the bsAb (DUPA-OKT3) and parental antibody (OKT3). On day 0, C4-2 cells (2 × 10^6^ cells) in 50% Matrigel (200 μL) were implanted s.c. (using a 25-gauge needle) in the right shoulder of 6- to 8-week-old male NCG mice. During the study, *ex vivo* expansion of T cells was started: Purified PBMCs were transferred to plates coated with anti-CD3 (PeproTech) and anti-CD28 (PeproTech) plates at 37 ˚C. After 2 days, the PBMCs were transferred into 10 cm dish and were incubated in RPMI medium supplemented with 10% FBS and 100 units/mL IL-2 (Genscript) for T-cell proliferation. On day 18, *ex vivo*-expanded T cells (25 × 10^6^ cells) were injected i.p. to augment T-cell response. Treatment was started on day 18 by i.v. injection of 1 mg/kg of DUPA-OKT3 or OKT3 for the treatment group and saline for the control group for 10 days. Tumor length, width, and height were monitored twice a week by caliper measurements, and the tumor volume was estimated as described above. Fifty microliters of murine blood were drawn at 24 h after the first drug dosing to determine serum IFN-γ concentrations using the ELISA kit (Thermo Fisher Scientific). All procedures were reviewed and approved by the Laboratory Animal Center of Peking University Shenzhen Graduate School, and were performed using protocols in accordance with the relevant guidelines and regulations.

## Supplementary Material

Supplementary methods and figures.

## References

[1] Agarwal P, Bertozzi CR (2015). Site-Specific Antibody-Drug Conjugates: The Nexus of Bioorthogonal Chemistry, Protein Engineering, and Drug Development. Bioconjugate Chem.

[2] Chudasama V, Maruani A, Caddick S (2016). Recent advances in the construction of antibody-drug conjugates. Nature Chemistry.

[3] Wu AM, Senter PD (2005). Arming antibodies: prospects and challenges for immunoconjugates. Nature Biotechnology.

[4] Fournier P, Schirrmacher V (2013). Bispecific antibodies and trispecific immunocytokines for targeting the immune system against cancer: preparing for the future. BioDrugs.

[5] Beck A, Goetsch L, Dumontet C, Corvaïa N (2017). Strategies and challenges for the next generation of antibody-drug conjugates. Nature Reviews Drug Discovery.

[6] Kline T, Steiner AR, Penta K, Sato AK, Hallam TJ, Yin G (2015). Methods to Make Homogenous Antibody Drug Conjugates. Pharm Res.

[7] Wang L, Amphlett G, Blättler WA, Lambert JM, Zhang W (2005). Structural characterization of the maytansinoid-monoclonal antibody immunoconjugate, huN901-DM1, by mass spectrometry. Protein Sci.

[8] Sun MMC, Beam KS, Cerveny CG, Hamblett KJ, Blackmore RS, Torgov MY (2005). Reduction-alkylation strategies for the modification of specific monoclonal antibody disulfides. Bioconjug Chem.

[9] Hamblett KJ, Senter PD, Chace DF, Sun MMC, Lenox J, Cerveny CG (2004). Effects of drug loading on the antitumor activity of a monoclonal antibody drug conjugate. Clin Cancer Res.

[10] Akkapeddi P, Azizi S-A, Freedy AM, Cal PMSD, Gois PMP, Bernardes GJL (2016). Construction of homogeneous antibody-drug conjugates using site-selective protein chemistry. Chem Sci.

[11] Jackson DY (2016). Processes for Constructing Homogeneous Antibody Drug Conjugates. Org Process Res Dev.

[12] Dai Z, Zhang X-N, Nasertorabi F, Cheng Q, Li J, Katz BB (2020). Synthesis of site-specific antibody-drug conjugates by ADP-ribosyl cyclases. Sci Adv.

[13] Eldridge GM, Weiss GA (2011). Hydrazide Reactive Peptide Tags for Site-Specific Protein Labeling. Bioconjugate Chem.

[14] Nunes JPM, Morais M, Vassileva V, Robinson E, Rajkumar VS, Smith MEB (2015). Functional native disulfide bridging enables delivery of a potent, stable and targeted antibody-drug conjugate (ADC). Chem Commun.

[15] Junutula JR, Raab H, Clark S, Bhakta S, Leipold DD, Weir S (2008). Site-specific conjugation of a cytotoxic drug to an antibody improves the therapeutic index. Nature Biotechnology.

[16] Lee MTW, Maruani A, Baker JR, Caddick S, Chudasama V (2016). Next-generation disulfide stapling: reduction and functional re-bridging all in one. Chem Sci.

[17] Maruani A, Smith MEB, Miranda E, Chester KA, Chudasama V, Caddick S (2015). A plug-and-play approach to antibody-based therapeutics *via* a chemoselective dual click strategy. Nature Communications.

[18] Badescu G, Bryant P, Bird M, Henseleit K, Swierkosz J, Parekh V (2014). Bridging Disulfides for Stable and Defined Antibody Drug Conjugates. Bioconjugate Chem.

[19] Agarwal P, Weijden J van der, Sletten EM, Rabuka D, Bertozzi CR (2013). A Pictet-Spengler ligation for protein chemical modification. PNAS.

[20] Carrico IS, Carlson BL, Bertozzi CR (2007). Introducing genetically encoded aldehydes into proteins. Nature Chemical Biology.

[21] Hudak JE, Barfield RM, de Hart GW, Grob P, Nogales E, Bertozzi CR (2012). Synthesis of Heterobifunctional Protein Fusions Using Copper-Free Click Chemistry and the Aldehyde Tag. Angewandte Chemie International Edition.

[22] Boeggeman E, Ramakrishnan B, Pasek M, Manzoni M, Puri A, Loomis KH (2009). Site specific conjugation of fluoroprobes to the remodeled Fc N-glycans of monoclonal antibodies using mutant glycosyltransferases: application for cell surface antigen detection. Bioconjug Chem.

[23] Ramakrishnan B, Qasba PK (2002). Structure-based design of beta 1,4-galactosyltransferase I (beta 4Gal-T1) with equally efficient N-acetylgalactosaminyltransferase activity: point mutation broadens beta 4Gal-T1 donor specificity. J Biol Chem.

[24] Xiao H, Chatterjee A, Choi S, Bajjuri KM, Sinha SC, Schultz PG (2013). Genetic Incorporation of Multiple Unnatural Amino Acids into Proteins in Mammalian Cells. Angewandte Chemie International Edition.

[25] Axup JY, Bajjuri KM, Ritland M, Hutchins BM, Kim CH, Kazane SA (2012). Synthesis of site-specific antibody-drug conjugates using unnatural amino acids. Proc Natl Acad Sci U S A.

[26] Hoppmann C, Wang L (2019). Genetically encoding photoswitchable click amino acids for general optical control of conformation and function of proteins. Methods Enzymol.

[27] Wang N, Yang B, Fu C, Zhu H, Zheng F, Kobayashi T (2018). Genetically Encoding Fluorosulfate-l-tyrosine To React with Lysine, Histidine, and Tyrosine via SuFEx in Proteins in Vivo. J Am Chem Soc.

[28] Luo X, Fu G, Wang RE, Zhu X, Zambaldo C, Liu R (2017). Genetically encoding phosphotyrosine and its nonhydrolyzable analog in bacteria. Nature Chemical Biology.

[29] Rabuka D, Rush JS, deHart GW, Wu P, Bertozzi CR (2012). Site-specific chemical protein conjugation using genetically encoded aldehyde tags. Nature Protocols.

[30] Luo X, Fu G, Wang RE, Zhu X, Zambaldo C, Liu R (2017). Genetically encoding phosphotyrosine and its nonhydrolyzable analog in bacteria. Nat Chem Biol.

[31] Chen Y, Loredo A, Gordon A, Tang J, Yu C, Ordonez J (2018). A noncanonical amino acid-based relay system for site-specific protein labeling. Chem Commun (Camb).

[32] Saghatelian A, Jessani N, Joseph A, Humphrey M, Cravatt BF (2004). Activity-based probes for the proteomic profiling of metalloproteases. Proc Natl Acad Sci U S A.

[33] Liu Y, Patricelli MP, Cravatt BF (1999). Activity-based protein profiling: the serine hydrolases. Proc Natl Acad Sci U S A.

[34] Yang B, Tang S, Ma C, Li S-T, Shao G-C, Dang B (2017). Spontaneous and specific chemical cross-linking in live cells to capture and identify protein interactions. Nat Commun.

[35] Xiang Z, Ren H, Hu YS, Coin I, Wei J, Cang H (2013). Adding an unnatural covalent bond to proteins through proximity-enhanced bioreactivity. Nature Methods.

[36] Xuan W, Li J, Luo X, Schultz PG (2016). Genetic Incorporation of a Reactive Isothiocyanate Group into Proteins. Angew Chem Int Ed Engl.

[37] Furman JL, Kang M, Choi S, Cao Y, Wold ED, Sun SB (2014). A Genetically Encoded aza-Michael Acceptor for Covalent Cross-Linking of Protein-Receptor Complexes. J Am Chem Soc.

[38] Jessani N, Cravatt BF (2004). The development and application of methods for activity-based protein profiling. Curr Opin Chem Biol.

[39] Berdan VY, Klauser PC, Wang L (2021). Covalent peptides and proteins for therapeutics. Bioorganic & Medicinal Chemistry.

[40] Yu C, Tang J, Loredo A, Chen Y, Jung SY, Jain A (2018). Proximity-Induced Site-Specific Antibody Conjugation. Bioconjugate Chem.

[41] Tian Z, Wu L, Yu C, Chen Y, Xu Z, Bado I (2021). Harnessing the power of antibodies to fight bone metastasis. Science Advances.

[42] Wu K-L, Yu C, Lee C, Zuo C, Ball Z, Xiao H (2021). Precision Modification of Native Antibodies. Bioconjugate Chemistry.

[43] Deisenhofer J (1981). Crystallographic refinement and atomic models of a human Fc fragment and its complex with fragment B of protein A from Staphylococcus aureus at 2.9- and 2.8-.ANG. resolution. Biochemistry.

[44] Léonetti M, Thai R, Cotton J, Leroy S, Ducancel F, Boulain JC Increasing Immunogenicity of Antigens Fused to Ig-Binding Proteins by Cell Surface Targeting. The Journal of Immunology.: 9.

[45] Chan W, White P (1999). Fmoc Solid Phase Peptide Synthesis: A Practical Approach. Oxford University Press.

[46] Matysiak S, Böldicke T, Tegge W, Frank R (1998). Evaluation of monomethoxytrityl and dimethoxytrityl as orthogonal amino protecting groups in Fmoc solid phase peptide synthesis. Tetrahedron Letters.

[47] Chen H, Lin Z, Arnst KE, Miller DD, Li W (2017). Tubulin Inhibitor-Based Antibody-Drug Conjugates for Cancer Therapy. Molecules [Internet].

[48] Axup JY, Bajjuri KM, Ritland M, Hutchins BM, Kim CH, Kazane SA (2012). Synthesis of site-specific antibody-drug conjugates using unnatural amino acids. Proc Natl Acad Sci U S A.

[49] Li H, Yu C, Jiang J, Huang C, Yao X, Xu Q (2016). An anti-HER2 antibody conjugated with monomethyl auristatin E is highly effective in HER2-positive human gastric cancer. Cancer Biol Ther.

[50] Kularatne SA, Wang K, Santhapuram H-KR, Low PS (2009). Prostate-specific membrane antigen targeted imaging and therapy of prostate cancer using a PSMA inhibitor as a homing ligand. Mol Pharm.

[51] May KF, Gulley JL, Drake CG, Dranoff G, Kantoff PW (2011). Prostate cancer immunotherapy. Clin Cancer Res.

[52] Bühler P, Molnar E, Dopfer EP, Wolf P, Gierschner D, Wetterauer U (2009). Target-dependent T-cell activation by coligation with a PSMA x CD3 diabody induces lysis of prostate cancer cells. J Immunother.

[53] Friedrich M, Raum T, Lutterbuese R, Voelkel M, Deegen P, Rau D (2012). Regression of human prostate cancer xenografts in mice by AMG 212/BAY2010112, a novel PSMA/CD3-Bispecific BiTE antibody cross-reactive with non-human primate antigens. Mol Cancer Ther.

