# Supplementary material for: Synthesis of precision antibody conjugates using proximity-induced chemistry
Source: Theranostics. 2021 Aug 27;11(18):9107–17. doi: 10.7150/thno.62444 (PMC8419051; doi:10.7150/thno.62444)
Supplement: Supplementary file 1 — Supplementary methods and figures. [file thnov11p9107s1.pdf]

Synthesis of Precision Antibody Conjugates using Proximity-Induced Chemistry

Yu J. Cao[a, §, *], Chenfei Yu[b, §], Kuan-Lin Wu[b, §], Xuechun Wang[a], Dong Liu[a], Zeru Tian[b], Lijun Zhao[a], Xuexiu Qi[a], Axel Loredo[b], Anna Chung[c], and Han Xiao[b, c, d, *]

[a] State Key Laboratory of Chemical Oncogenomics, Key Laboratory of Chemical Genomics, Peking University Shenzhen Graduate School, Shenzhen, 518055, China
[b] Department of Chemistry, Rice University, 6100 Main Street, Houston, Texas, 77005, USA
[c] Department of Biosciences, Rice University, 6100 Main Street, Houston, Texas, 77005, USA
[d] Department of Bioengineering, Rice University, 6100 Main Street, Houston, Texas, 77005, USA

§ These authors contributed equally
* To whom correspondence should be addressed: joshuacao@pku.edu.cn; han.xiao@rice.edu

Chemical synthesis

Synthesis of DBCO-PEG4-pNP

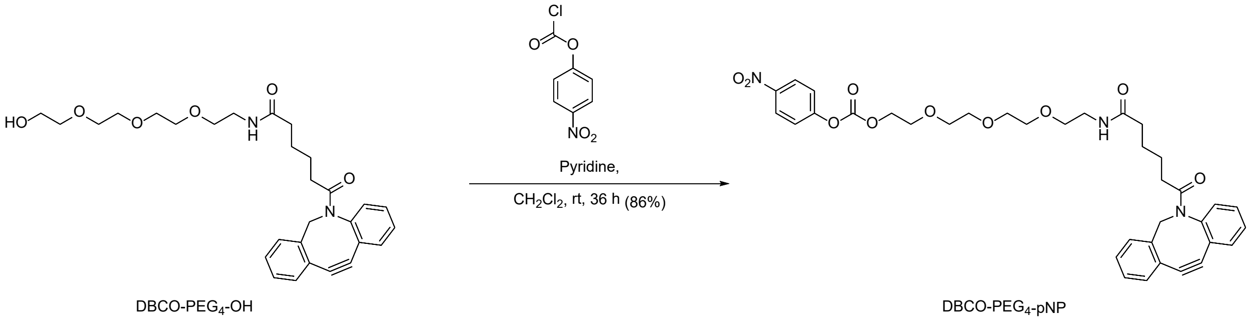

DBCO-PEG4-OH (12 mg, 0.024 mmol), pyridine (6 uL, 0.075 mmol) 4-Nitrobenzyl chloroformate (5.3 mg, 0.026 mmol) were stirred in DCM (3 mL) for 36 at room temperature, under nitrogen. The solvent was evaporated under reduced pressure. The residue was purified by HPLC, the pure fractions were subjected to lyophilization to afford a yellow oil (14 mg obtained, 86%) ESI-MS $[M+H]^+$ calcd. for $C_{36}H_{39}N_3O_{10}$ 674.3, found 674.3.

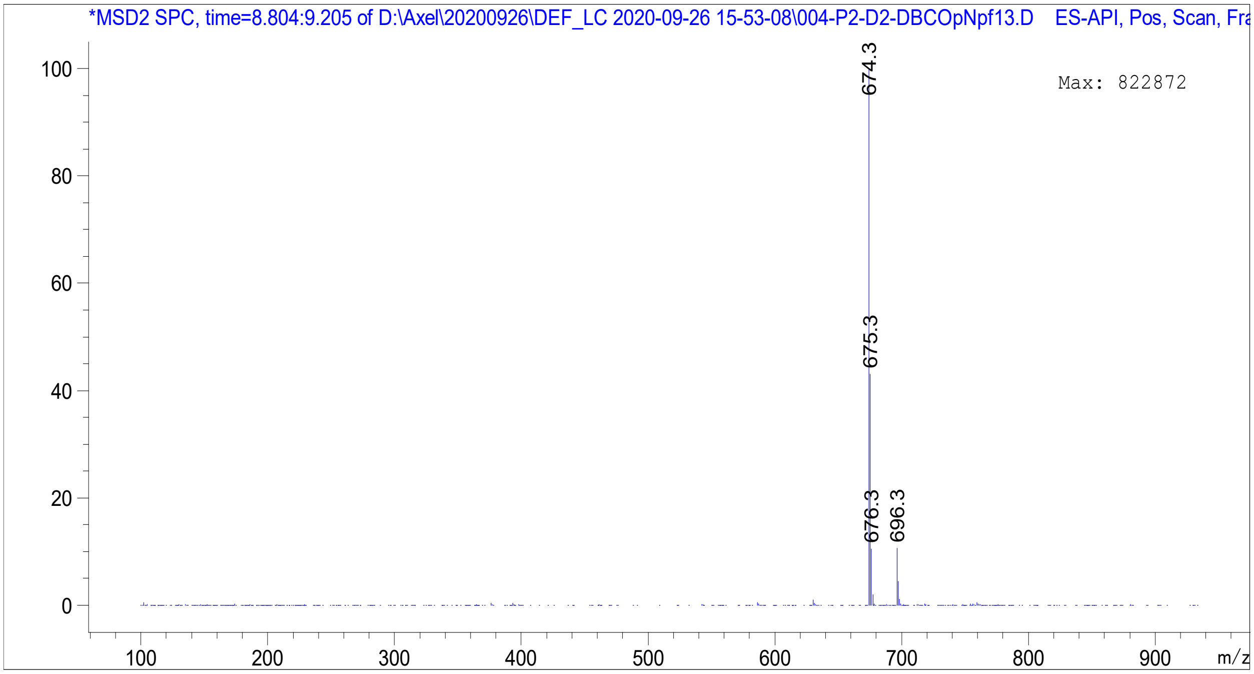

Synthesis of DBCO-PEG4-MMAE

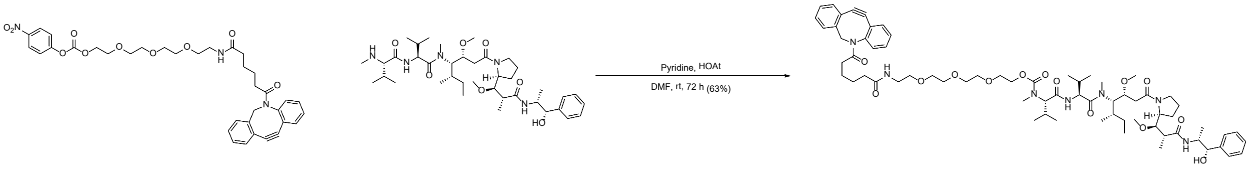

DBCO-PEG-4-pNP (6.7 mg, 0.01 mmol) pyridine (3 uL, 0.036 mmol), HOAt (1 mg, 0.01 mmol) and MMAE (8 mg, 0.011 mmol) were dissolved in DMF (2 mL). The resulting solution was stirred for 72 h under nitrogen at room temperature. The solvent was evaporated under reduced pressure. The residue was purified by HPLC, the pure fractions were subjected to lyophilization to afford a white powder (8.5 mg, 63%) ESI-MS $[M+H]^+$ calcd. for $C_{69}H_{101}N_7O_{14}$ 1253.7, found 1253.7. $[M+H]^{2+}$ calcd. 627.3, found 627.0.

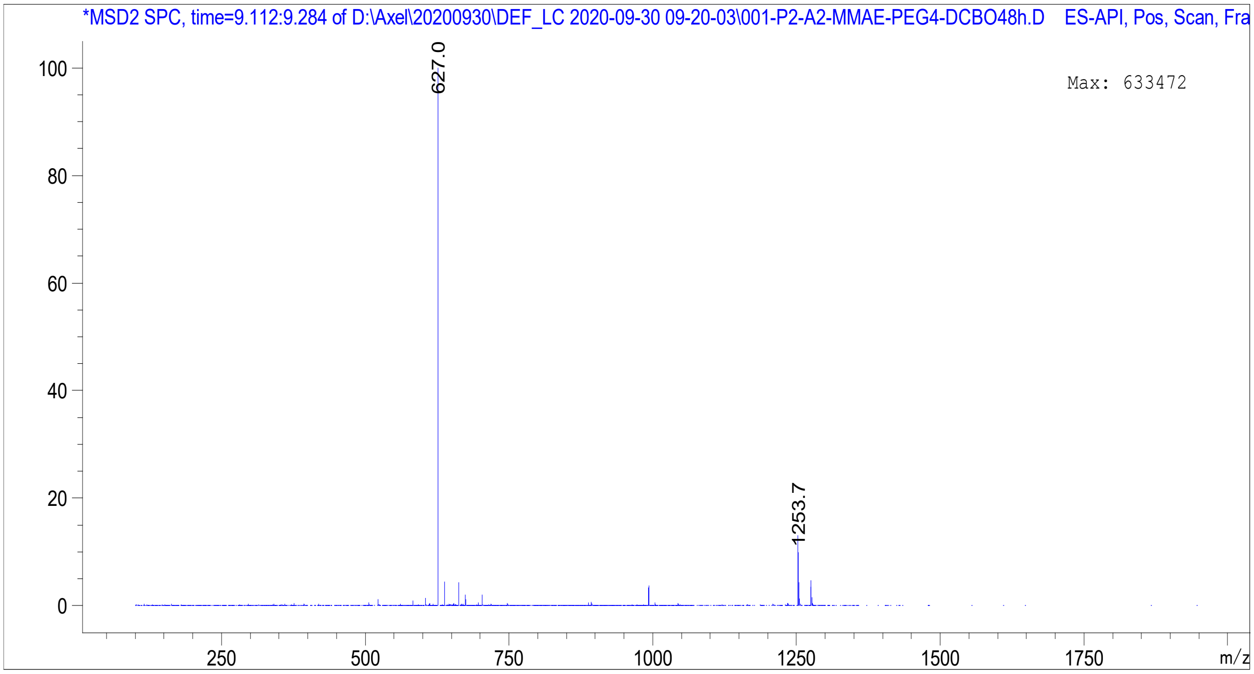

Synthesis of BCN-DUPA

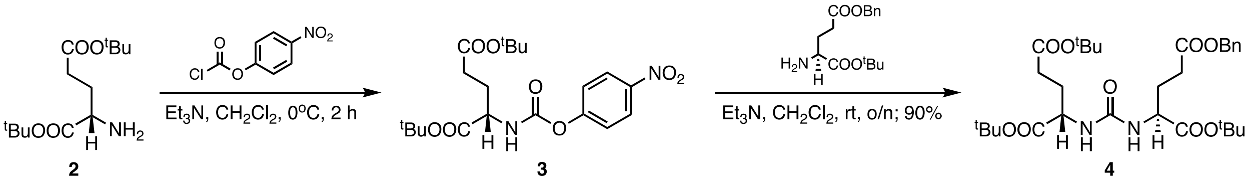

L-glutamic acid di-tert-butyl ester hydrochloride (500 mg, 1.69 mmol, 1 equiv.), 4-nitrophenyl chloroformate (375 mg, 1.86 mmol, 1.1 equiv.) was dissolved in dry CH_2Cl_2 (20 mL) under N_2 protection. The reaction mixture was cooled down by ice bath, and triethylamine was slowly added (513 mg, 0.7 mL, 5.07 mmol, 3 equiv.). After stirring for 2 h, the reaction mixture was extracted by 0.1 N $HCl_{(aq)}$ (3 x 20 mL), dried over $MgSO_4$, evaporated under reduced pressure. Without further purification, the crude product along with *L*-glutamic acid 1-(1,1-dimethylethyl) 5-(phenylmethyl) ester hydrochloride (613 mg, 1.86 mmol, 1.1 equiv.) was dissolved in dry CH_2Cl_2 (20 mL). After adding triethylamine (513 mg, 0.7 mL, 5.07 mmol, 3 equiv.), the mixture was stirred overnight at room temperature. The solvent was evaporated under reduced pressure, and the residual solid was redissolved into 20 mL EtOAc and washed with 1N Na_2CO_3 multiple times until the yellow side product was fully removed from the organic phase. The transparent organic phase was then washed with brine, dried over $MgSO_4$, and then evaporated under

reduced pressure to yield a colorless syrup **4** (880 mg; 90%). For calculated $[M+H]^+$ = 579.3, found $[M+H]^+$ = 579.3.

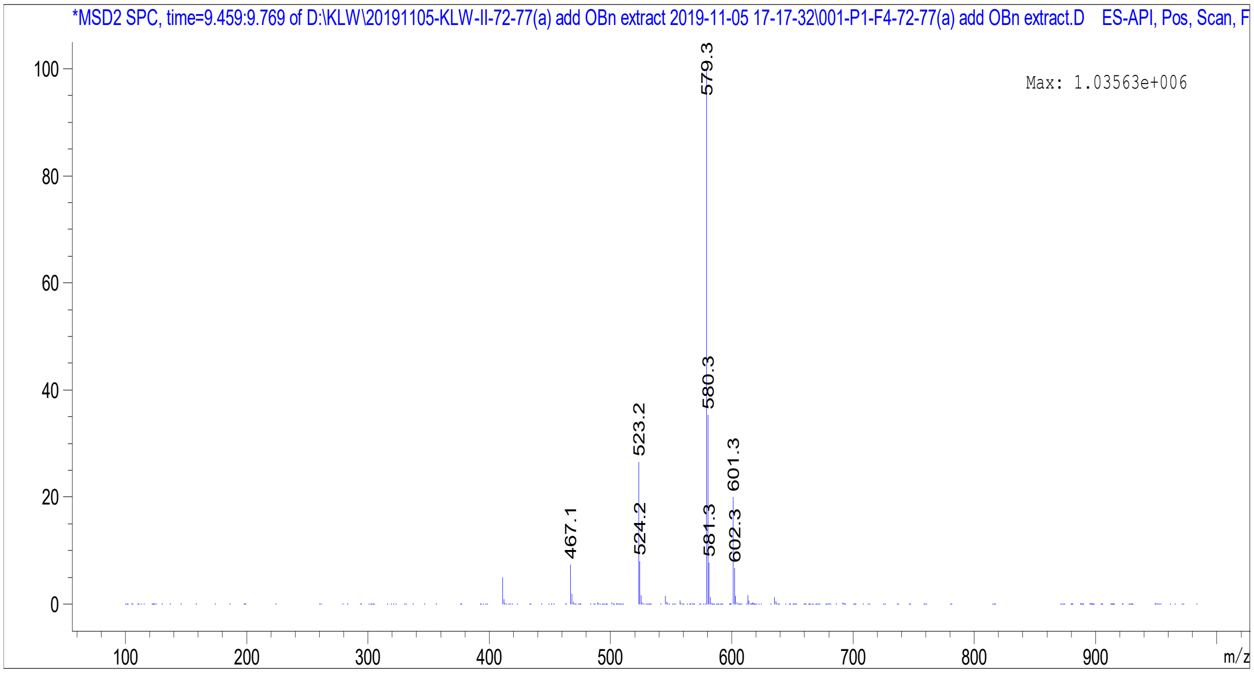

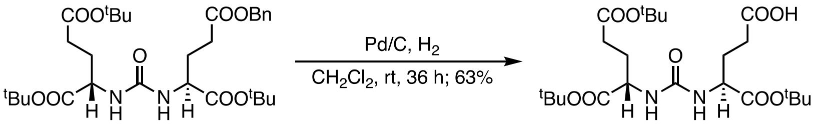

Compound **4** (300 mg, 0.52 mmol) was dissolved in CH_2Cl_2 (10 mL) and the round bottom flask was degassed with N_2 before palladium on carbon (Pd/C, 40 mg) was added to prevent Pd/C from being ignited. The mixture was hydrogenated at room temperature with a hydrogen balloon for 36 h and was then filtered and washed with EtOAc through a Celite pad. The crude product was purified by column chromatography (50% EtOAc in hexanes) to yield compound **5** as a colorless syrup (160 mg; 63%). For calculated $[M+H]^+$ = 489.3, found $[M+H]^+$ = 489.2.

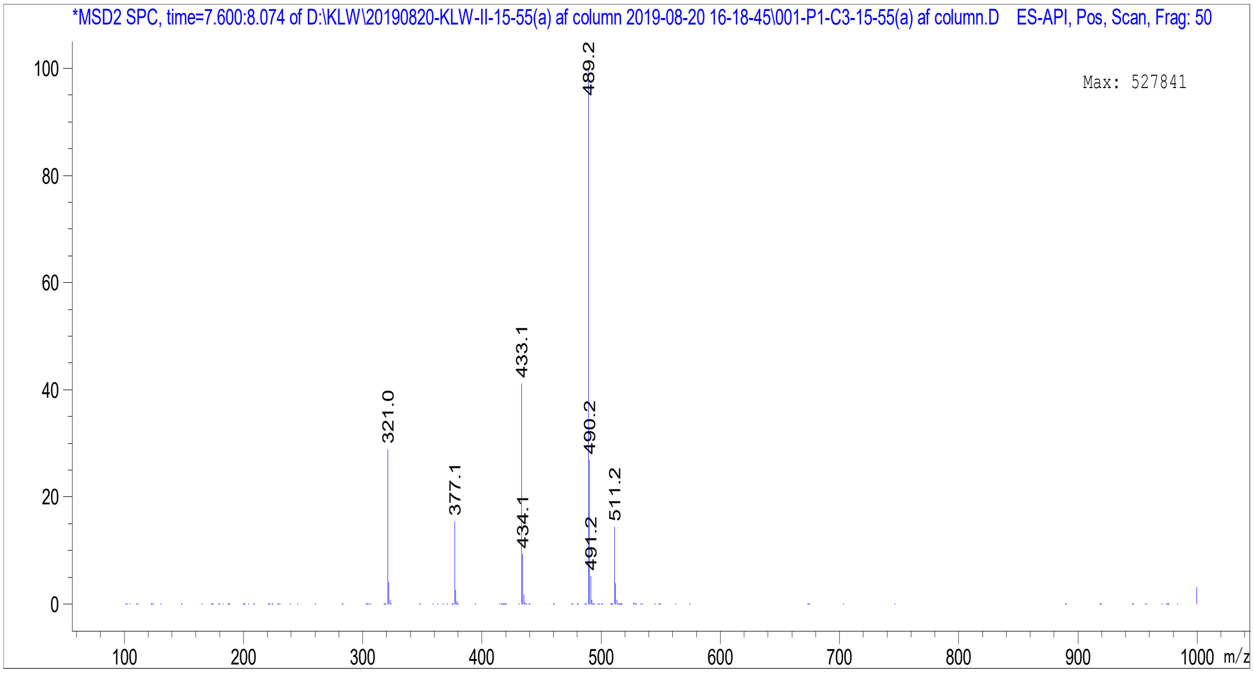

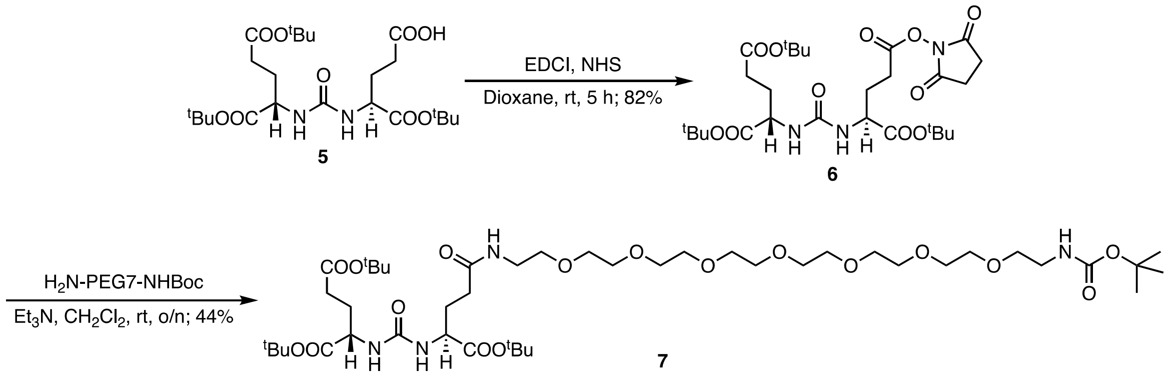

Compound **5** (160 mg, 0.328 mmol, 1 equiv.), 1-ethyl-3-(3-dimethylaminopropyl) carbodiimide HCl salt (EDC, 125.6 mg, 0.655 mmol, 2 equiv.), N-hydroxysuccinimide (NHS, 83 mg, 0.721 mmol, 2.2 equiv.) was dissolved in dry dioxane and stirred at room temperature for 5 h. After the solvent was evaporated, the mixture was redissolved in EtOAc (10 mL), extracted with saturated $NaHCO_{3(aq)}$ (3 x 5 mL), 1 N $HCl_{(aq)}$ (3 x 5 mL), brine (3 x 5 mL), dried over $MgSO_4$, and then evaporated under reduced pressure to yield a white solid **6** (158 mg; 82 %). Without further purification, compound **6** (56.8 mg, 0.097 mmol, 1 equiv.), t-boc-N-amido-PEG7-amine (50 mg, 0.107 mmol, 1.1 equiv.) was dissolved in CH_2Cl_2 (3 mL). After adding triethylamine (20 mg, 0.0276 mL, 0.198 mmol, 2 equiv.), the mixture was stirred overnight at room temperature. The reaction mixture was dried under reduced pressure, and the resulting crude product was purified by column chromatography (5 % MeOH in EtOAc) to yield compound 7 as a colorless oil (50 mg; 55 %). For calculated $[M+H]^+$ =939.6, found $[M+H]^+$ = 939.5.

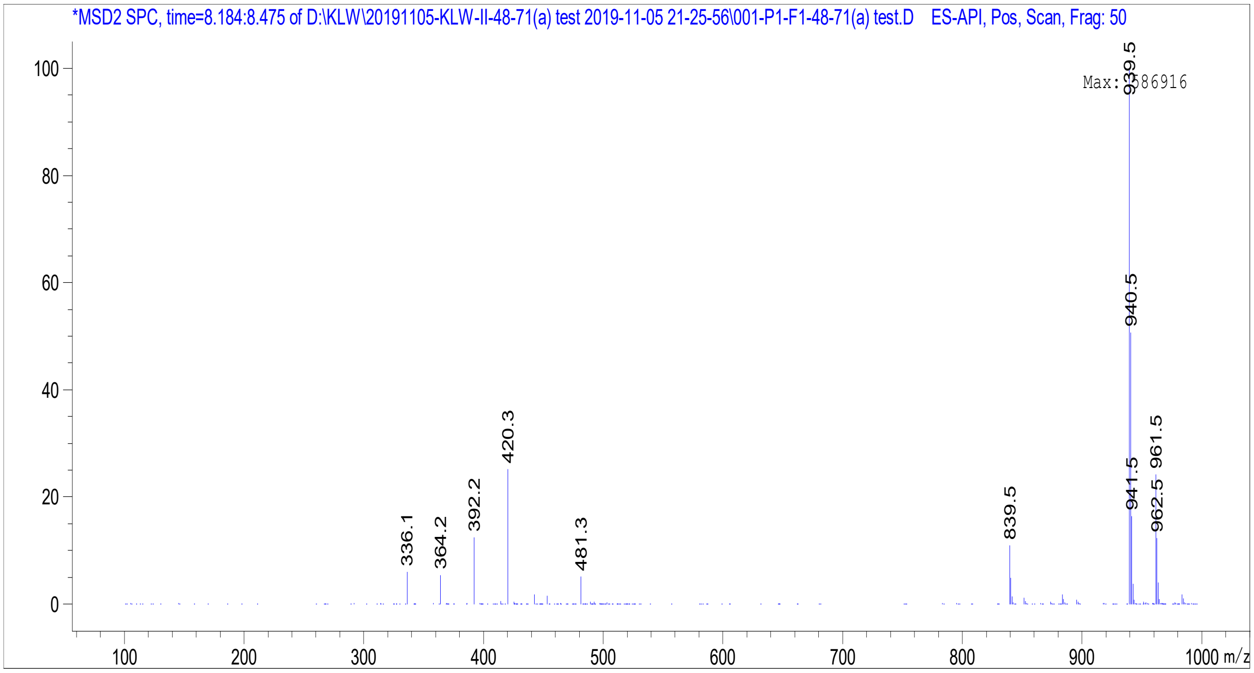

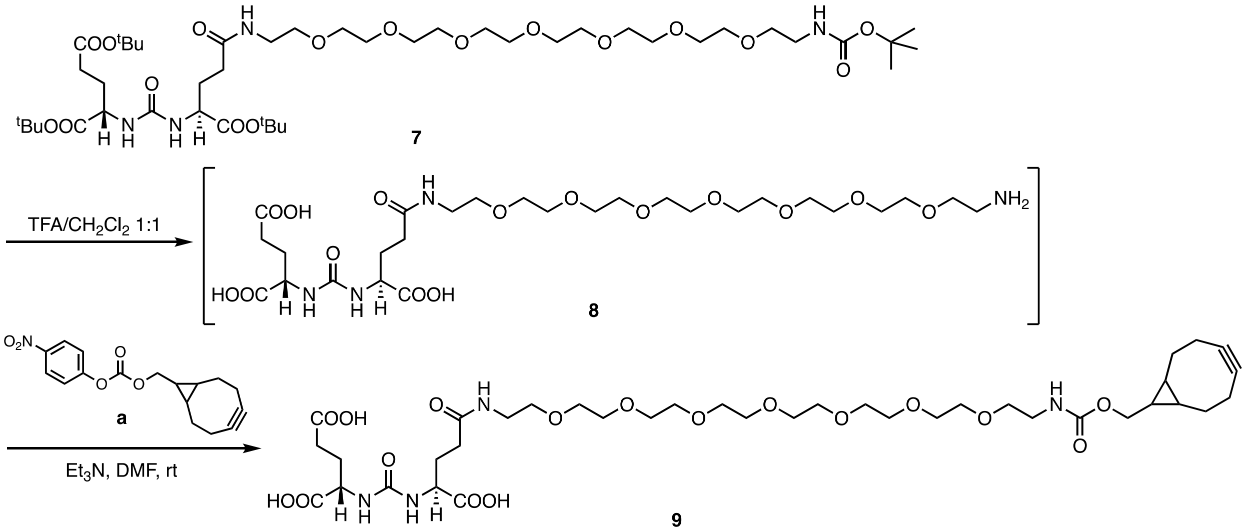

Compound **7** (50 mg, 0.053 mmol, 1 equiv.) was dissolved in CH_2Cl_2 (1 mL). After cooling down the reaction in iced bath, trifluoroacetic acid (1 mL) was added dropwise and then the reaction was stirred at room temperature for 5 h. After the reaction was completely dried, BCN-PNP **a** (20 mg, 0.64 mmol, 1.2 equiv.), dimethylformamide (DMF, 2 mL), triethylamine (32.2 mg, 0.045 mL, 0.318 mmol, 6 equiv.) was added and the reaction was stirred at room temperature for 1 h. The solvent was evaporated, and the crude product was purified by purified HPLC (5% MeCN to 100% MeCN in H_2O) to yield the final BCN-DUPA **9** as a colorless oil (4.5 mg; 10%). For calculated $[M+H]^+$ =847.4, found $[M+H]^+$ = 847.4.

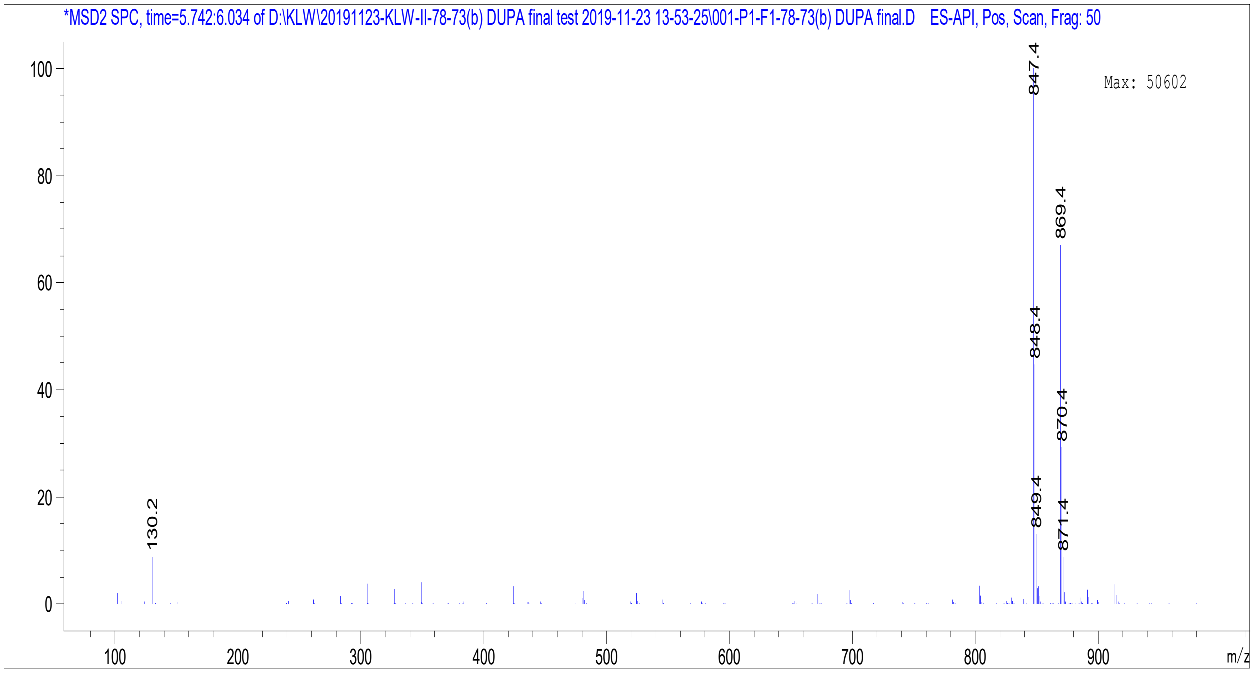

^{1}H NMR (600 MHz, D_2O)

Molecular Weight: 846.93

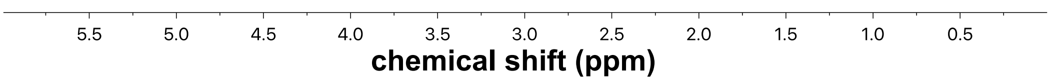

^{13}C NMR (150 MHz, D_2O)

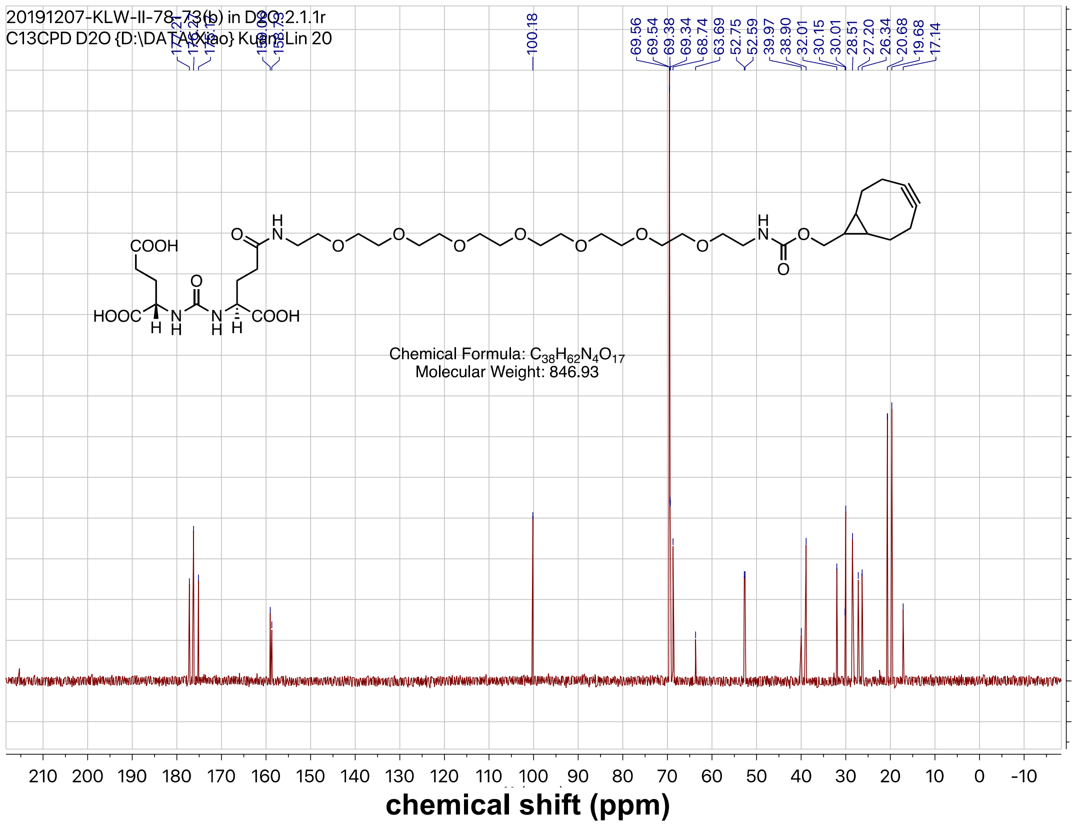

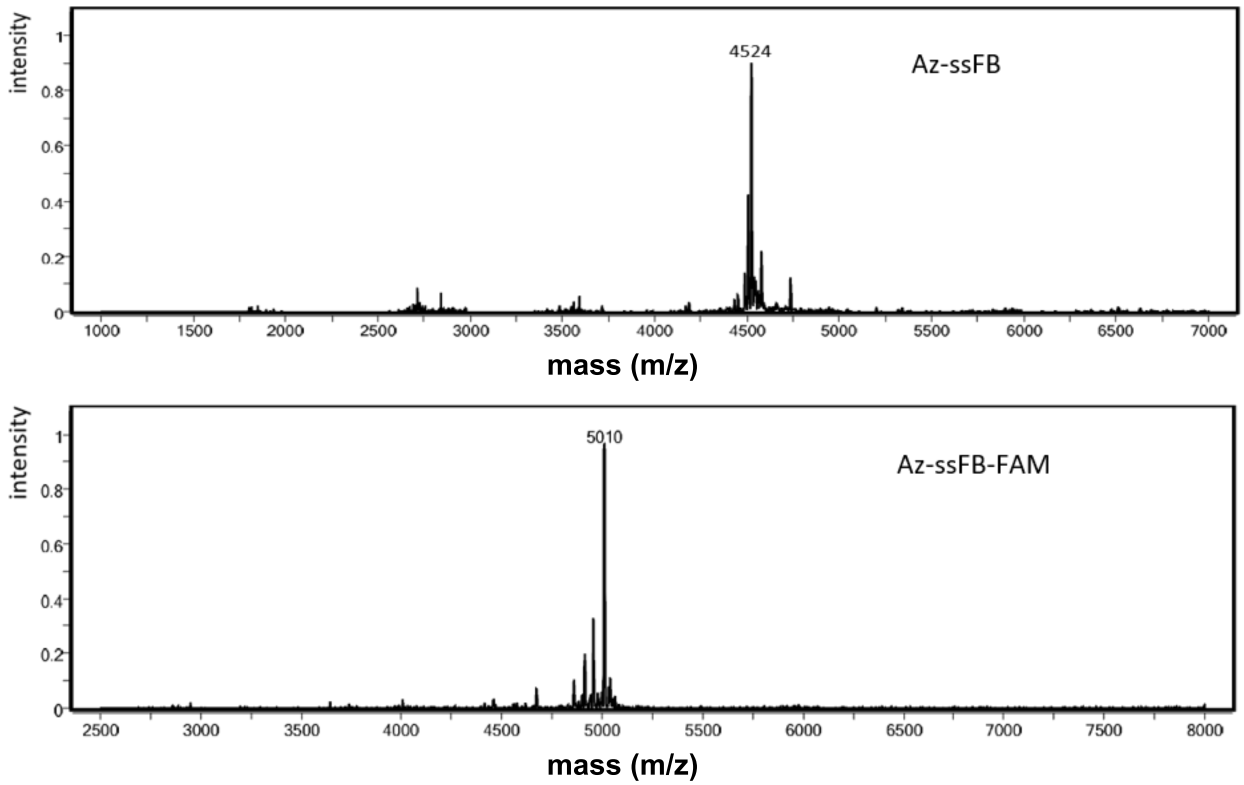

Figure S1. ESI-MS analysis of Az-ssFB and Az-ssFB-FAM.

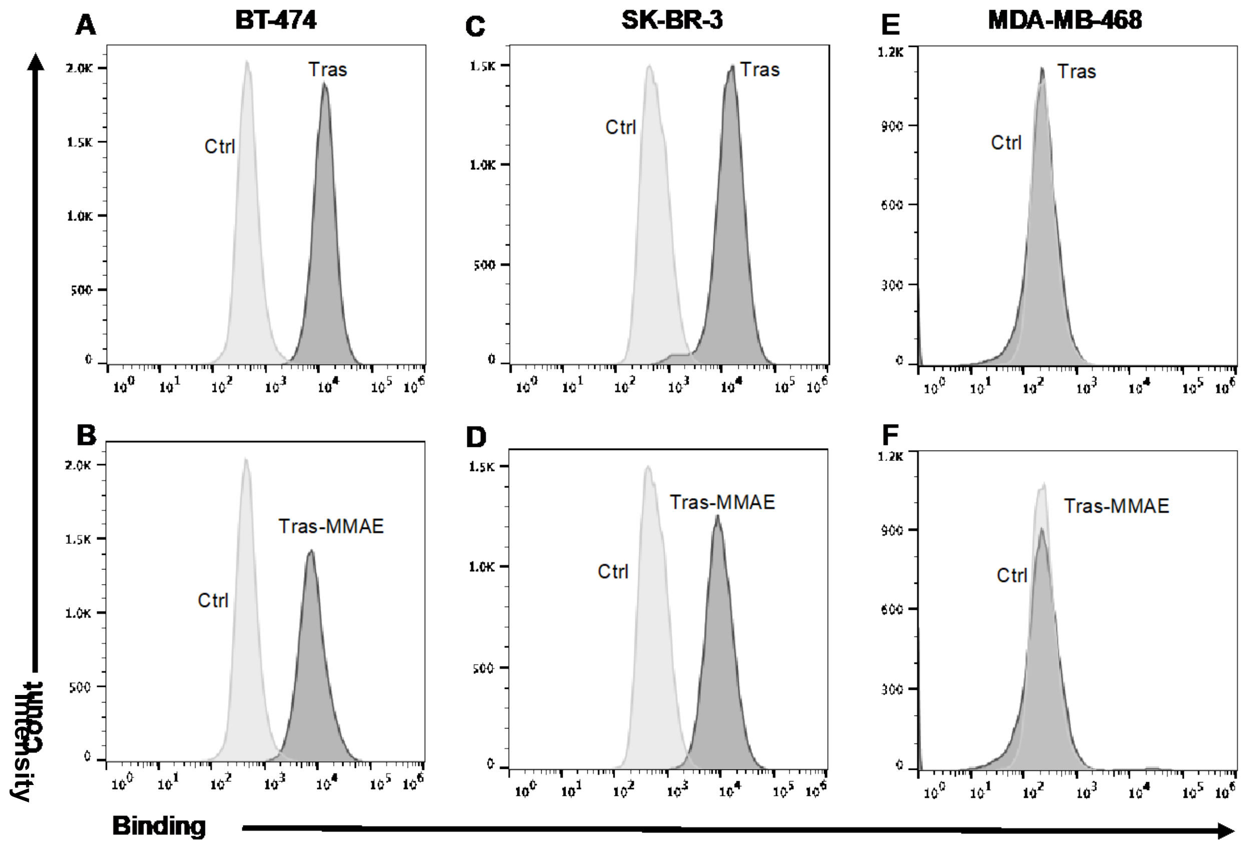

Figure S2. Flow cytometry analysis of receptor binding of Tras and Tras-MMAE to (A, B) HER2+ BT474, (C, D) HER2+ SK-BR-3 and (E, F) HER- MDA-MB-468 cell line. Staining conditions: Tras-Auristatin-FITC was incubated with cells at 1 µg/million cells on ice for 30 min.

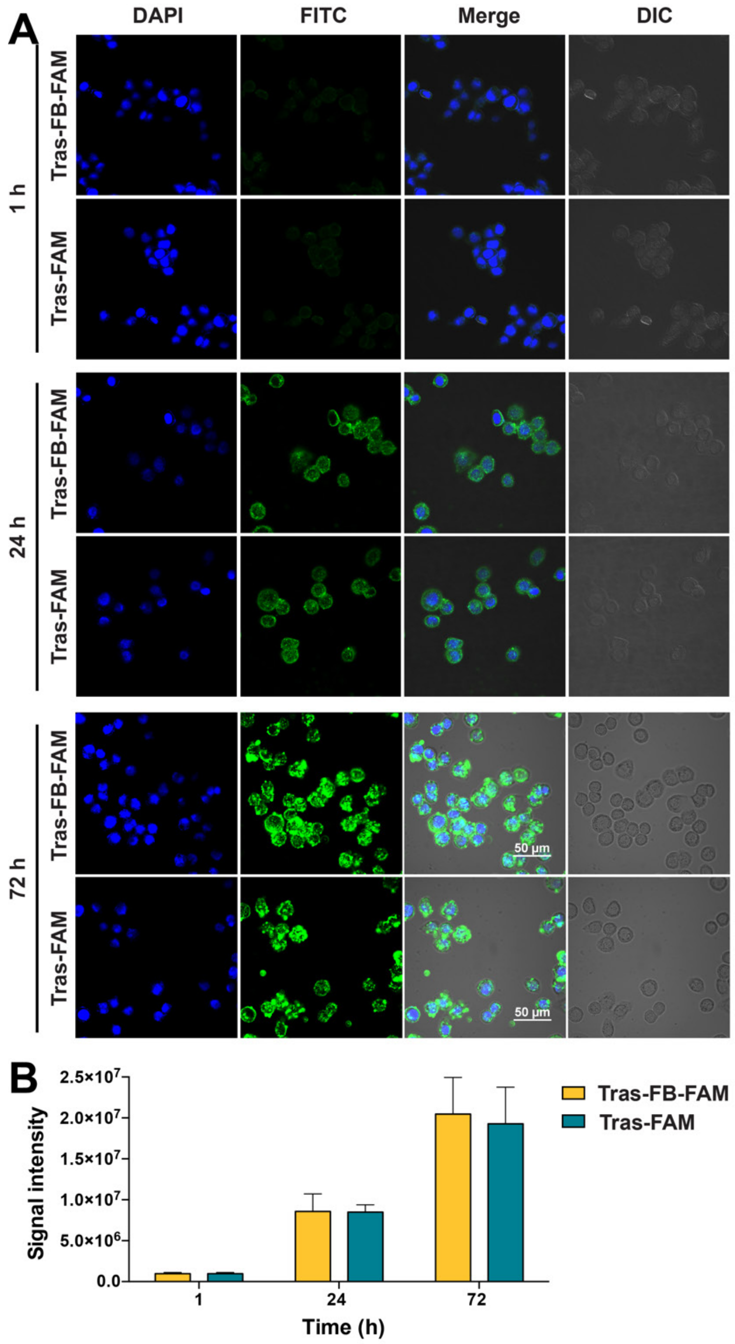

Figure S3. (A) The internalization of Tras-FAM and Tras-FB-FAM was monitored using fluorescent imaging. SK-BR-3 cells were incubated with 10 nM Tras-FAM and Tras-FB-FAM for 1, 24 and 72 hours and stained with Hoechst stain (Blue). The cells were washed twice with PBS and incubated with glycine buffer (0.5M NaCl, 0.1M glycine, pH2.5) to remove the antibody bound to the cell surface. Cells were then washed three times with PBS (pH 7.4) and used for confocal imaging. Scale bar = 50 μm. **(B)** Signal intensities of FAM inside of the cell have been measured to quantify the uptake efficiency.

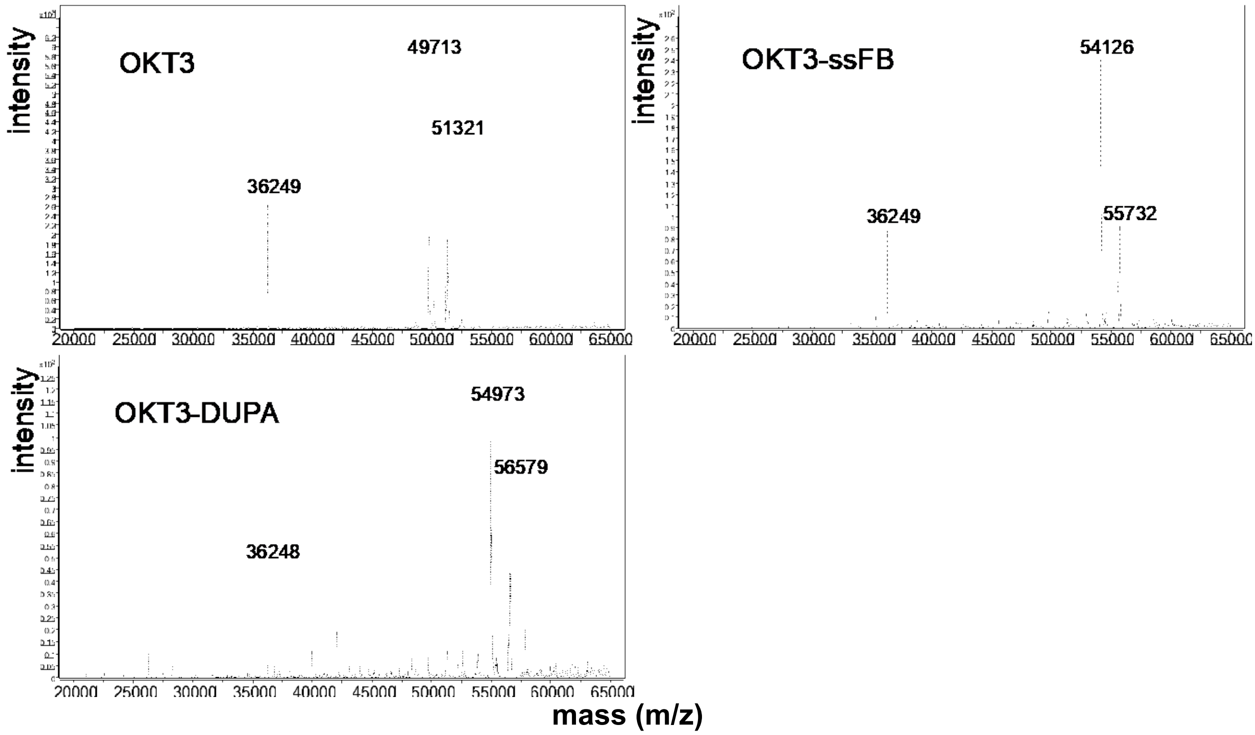

Figure S4. ESI-MS analysis of OKT3 and DUPA-OKT3 antibodies.

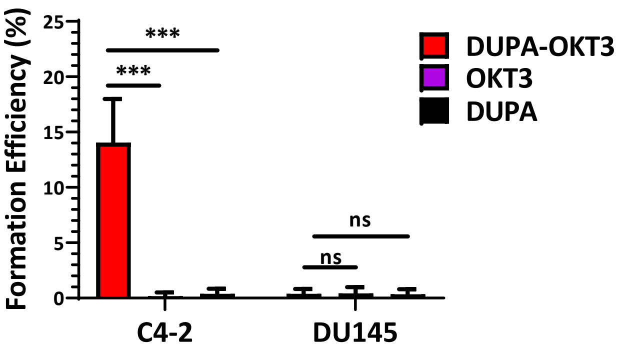

Figure S5. Fluorescence microscopy images of the interaction between C4-2 (red) cells and Jurkat cells (green) in the presence of the DUPA, OKT3 and the DUPA-OKT3 conjugate. The bar graphs were calculated and expressed as mean ± SD (n > 10).

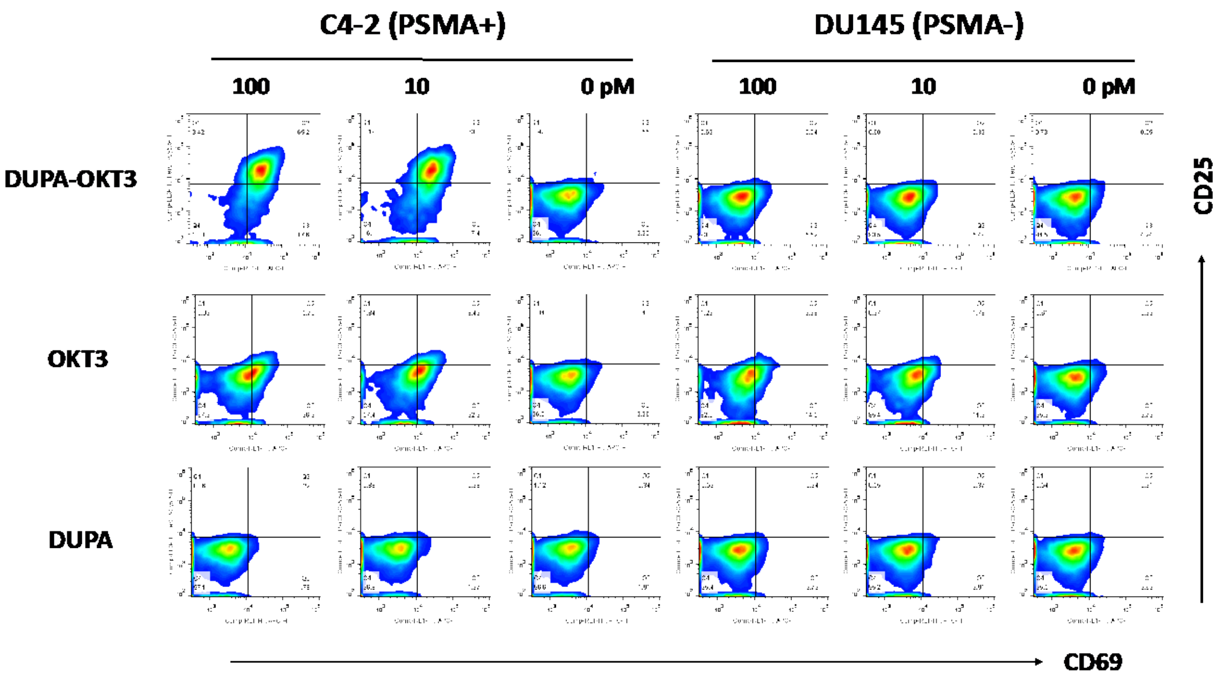

Figure S6. Flow cytometry analysis of T cell activation by DUPA-OKT3, OKT3 and DUPA against PSMA-positive C4-2 cancer cell and PSMA-negative DU145 cancer cell. T cells were cocultured with cancer cells at E: T = 5: 1 with 100, 10 or 0 pM of the different drugs for 24h. T cell activation was evaluated by flow cytometry with staining for CD69 and CD25 expression.

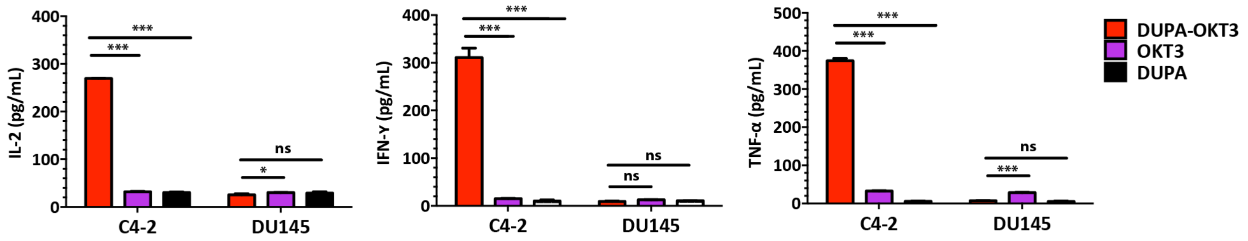

Figure S7. T cell activation by DUPA-OKT3, OKT3 and DUPA against PSMA-positive C4-2 cancer cell and PSMA-negative DU145 cancer cell. T cells were cocultured with cancer cells at E: T = 5: 1 with 100 pM of the different drugs for 24h. IL-2, IFN-γ and TNF-α levels from the incubation medium were measured by ELISA kit. Error bars represent standard deviation of duplicate samples.

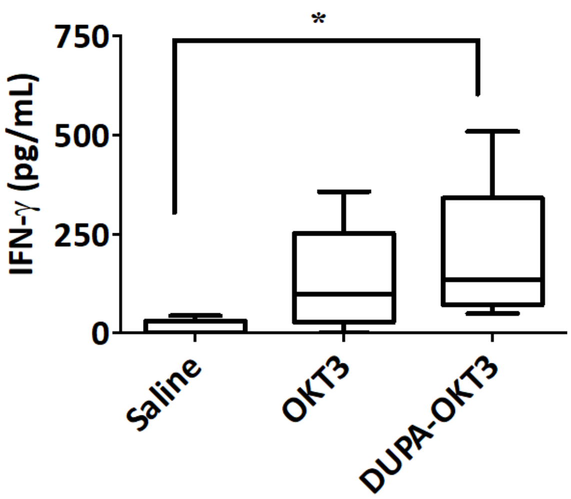

Figure S8. Serum Level of IFN-γ at 24h after first dose, from groups dosed every two days with saline, OKT3 and DUPA-OKT3 (n=5). The IFN-γ was determined by ELISA kit. Error bars represent standard deviation of five samples, and error bars represent SD. *=P < 0.05 was calculated using the Student's t test.
